# CD19-targeted HSP90 inhibitor nanoparticle combined with TKIs reduces tumor burden and enhances T-cell immunity in murine B-cell malignancies

**DOI:** 10.7150/thno.106758

**Published:** 2025-02-25

**Authors:** Mengting Qin, Juan Ren, Xiaodong Chen, Wen Zhou, Shuyuan Zhang, Weile Zhang, Mengxin Shi, Mingzhen Zhang, Huashen Liu, Yunfeng Ma, Mei Yang, Yanhong Ji

**Affiliations:** 1 Department of Pathogenic Biology and Immunology. School of Basic Medical Sciences, Xi'an Jiaotong University. Xi'an, Shaanxi, 710061, China.; 2 Department of Hematology, the First Affiliated Hospital of Xi'an Jiaotong University. Xi'an, Shaanxi, 710061, China.; 3 Department of Biophysics, School of Basic Medical Sciences, Xi'an Jiaotong University. Xi'an, Shaanxi, 710061, China.; 4 Department of Organ Procurement and Allocation, the First Affiliated Hospital of Xi'an Jiaotong University, Xi'an, Shaanxi, 710061, China.

**Keywords:** BCR-ABL1^+^ B-ALL, B-cell lymphomas, PLGA, HSP90, Anti-CD19 scFv, T cell immune response, MHC-I

## Abstract

**Rationale:** Conventional chemotherapies for B-cell malignancies are often limited by drug resistance and significant side effects due to non-specific targeting. This research aimed to improve treatment efficacy by developing nano-delivery systems that specifically target tumor cells, thereby enhancing therapeutic precision and reducing off-target toxicity.

**Methods:** The construction, biocompatibility, and targeting capability of CD19@NP/17-DMAG were evaluated using TEM, HPLC, FTIR spectroscopy, CCK-8 assay, flow cytometry (FC), and IVIS imaging. Therapeutic efficacy was assessed through Western blotting, RT-qPCR, flow cytometry, H&E staining, BrdU assay, and apoptosis assays. The mechanism of action of CD19@NP/17-DMAG in murine B-cell malignancies was investigated using RNA sequencing, *in vivo* T-cell depletion, and CRISPR/Cas9 technology.

**Results:** CD19@NP/17-DMAG nanoparticles demonstrated enhanced efficacy in murine models of BCR-ABL1⁺ B-cell acute lymphoblastic leukemia (B-ALL) when combined with tyrosine kinase inhibitors (TKIs), including the BCR-ABL1-targeted imatinib and the broad-spectrum ponatinib. This combination significantly reduced tumor burden, prolonged survival, and induced a robust anti-tumor T-cell response. RNA-seq analysis indicated that the targeted treatment modulated genes related to cell proliferation, apoptosis, and antigen presentation. Notably, this treatment also increased MHC class I (MHC-I) expression, thereby strengthening antigen presentation in BCR-ABL1⁺ B-ALL cells. Ponatinib-based therapy achieved complete remission, eradicated minimal residual disease, and established long-term immune memory in BCR-ABL1⁺ B-ALL. In addition, CD19@NP/17-DMAG was effective in another B-cell malignancy model, A20 lymphoma, significantly slowing tumor growth and amplifying T-cell responses.

**Conclusions:** These findings highlight the CD19@NP/17-DMAG system as a promising therapeutic approach that both augments T cell immune responses and minimizes side effects in B-cell malignancies.

## Introduction

B-cell malignancies account for the majority of hematological cancers, affecting over 500,000 people worldwide each year. Despite advancements in chemotherapies including small-molecule tyrosine kinase inhibitors (TKIs), targeted therapies such as monoclonal antibodies, and immunotherapies like chimeric antigen receptor (CAR-T) cells for B-cell leukemias such as BCR-ABL1^+^ B-cell acute lymphoblastic leukemia (BCR-ABL1^+^ B-ALL) and B-cell lymphomas, the 5-year survival rate for many B-cell malignancies remains below 70% 1-4. Furthermore, both conventional chemotherapy and targeted therapies carry significant risks, including severe myelosuppression, long-term organ damage, cytokine release syndrome and neurotoxicity 5-9. Consequently, the search for innovative strategies that achieve better efficacy with fewer side effects continues.

A large body of evidence has established CD19 as an ideal ligand for targeting B-cell malignancies, largely due to its role as a transmembrane protein predominately expressed on normal and malignant B lymphocytes. Functionally, CD19 is indispensable for B-cell activation and maturation, modulating signal transduction pathways that regulate both physiological and pathological immune responses. In tumor settings, the restricted expression of CD19 on B cells has been harnessed for therapeutic gain. CD19-targeted interventions, including monoclonal antibodies and CAR T-cell therapies, have demonstrated remarkable efficacy in reducing leukemia and lymphoma burden and improving patient outcomes 10-13. However, recent developments underscore the need for careful refinement of CD19-targeted approaches: some CD19 CAR T cells have been linked to severe or fatal cardiac events, whereas CD19/CD3 bispecific T-cell engagers can induce fatal hypotension in pediatric ALL 14. Conversely, engineering CD19 CAR T cells with hypoxia-controlled IL-12 may offer a safer, more targeted modality for treating diffuse large B-cell lymphoma (DLBCL) 15. Taken together, these observations underscore CD19's substantial promise as a selective and efficacious therapeutic target, reinforcing its value as a ligand in cutting-edge drug delivery platforms.

HSP90 has emerged as a highly promising target for cancer therapy. This molecular chaperone supports the function of more than 200 proteins that drive cancer progression 16, 17. Overexpression of HSP90 in cancer cells promotes oncoprotein maturation and accelerates cell growth 18, 19. By inhibiting HSP90, key cellular processes are disrupted, leading to protein degradation, elevated tumor antigen expression, enhanced MHC-I presentation, and increased inflammatory cytokine production 20-22. Moreover, by targeting oncogenic client proteins such as BCR-ABL1, HSP90 inhibitors enhance the effectiveness of TKIs, leading to apoptosis and potentially overcoming drug resistance in chronic myeloid leukemia (CML) and BCR-ABL1⁺ B-ALL. 20, 22-25. Beyond their antitumor effects, TKIs reinforce immune responses by reshaping the tumor microenvironment, thereby reactivating and restoring NK cell and T-cell surveillance in leukemia patients. Meanwhile, HSP90 inhibitors like 17-DMAG can promote immunogenic cell death and enhance antigen presentation, thus amplifying T-cell-mediated tumor eradication 26. Taken together, these immunomodulatory properties suggest that combining TKIs with HSP90 inhibitors may yield synergistic antitumor T-cell responses. Nonetheless, despite these advantages, the clinical application of HSP90 inhibitors remains constrained by significant toxicity and poor bioavailability 19.

Nanoparticle-based drug delivery systems have shown potential in improving cancer drug targeting, reducing toxicity, and increasing effectiveness 27-29. Poly (D, L-lactide-co-glycolide) (PLGA), an FDA-approved polymer, is highly biodegradable and offers controlled, sustained drug release 30-32. Surface modification of PLGA with poly (D, L-lactic acid)-polyethylene glycol-N-hydroxysuccinimide (PLA-PEG-NHS) further improves the functionality of nanoparticles by increasing their circulation time and reducing immune system recognition 32-35. Additionally, the NHS group enables stable bonding with proteins, allowing specific targeting of cancer cells 36, 37.

Here, we demonstrate that CD19-targeted HSP90 inhibitor 17-DMAG nanoparticle combined with TKI not only targets cancer cells effectively but also triggers an antitumor T-cell immune response in murine models of BCR-ABL1^+^ B-ALL and B-cell lymphoma. It significantly reduces tumor burden, prolongs survival, and may overcome the limitations of existing treatments. Our study supports this novel approach as a promising strategy for treating B-cell malignancies.

## Materials and Methods

### Materials

Poly (D, L-lactide-co-glycolide) (50:50) (PLGA) (Mw: 38,000-54,000) and poly (D, L-lactic acid)-polyethylene glycol-N-hydroxysuccinimide (PLA-PEG-NHS) (Mw: 15,000) were purchased from Sigma-Aldrich (St. Louis, MO, USA). The hydrochloride salt of alvespimycin (17-DMAG) (S1142), imatinib (S2475), and ponatinib (S1490) were obtained from Selleck Chemicals (Houston, TX, USA). The prokaryotic expression vector pMCSG7 was acquired from Ke Lei Biological Technology (Shanghai, China). Escherichia coli DH5α and BL21 (DE3) pLysS strains (Tiangen Biotech, Beijing, China) were used as cloning and protein expression hosts, respectively. Lipophilic dyes 1,1'-dioctadecyl-3,3,3',3'-tetramethylindotricarbocyanine iodide (DiR) (D4006) and 1,1'-dioctadecyl-3,3,3',3'-tetramethylindocarbocyanine perchlorate (DiL) (D4053) were purchased from UElandy (Beijing, China). Ovalbumin (OVA) peptide SIINFEKL (HY-P1489) was derived from MCE (New Jersey, USA).

### Preparation of nanoparticles

PLGA and PLGA-NHS complexed nanoparticles were synthesized using a single-step surface-functionalizing technique as previously described 38. The co-solvent dichloromethane (DCM) was removed by rotary evaporation. The emulsion was stirred for 3 h at room temperature, washed three times with deionized water, and then PLGA-NHS nanoparticles were collected by centrifugation (14,000 rpm, 15 min, 4°C). Control PLGA nanoparticles were also prepared under similar conditions. Both PLGA and PLGA-NHS were vacuum-dried to powder form and stored at -20°C.

### Characterization of nanoparticles

The morphology of PLGA and PLGA-NHS nanoparticles was examined using transmission electron microscopy (TEM). The diameter and zeta potential of nanoparticles were determined using a Malvern Zetasizer Nano ZS90 Apparatus (Malvern Instruments, UK) at room temperature.

The preparation method for 17-DMAG-loaded PLGA-NHS was identical to those used in the preparation of non-loaded nanoparticles. The entrapment efficiency and loading capacity of 17-DMAG were measured using an LC-2030 Plus high-performance liquid chromatography (HPLC) system (SHIMADZU, Japan) equipped with an Inertsil® ODS-3 column (5 μm, id 4.6 × 150 mm; SHIMADZU, Japan). The following formulas were used for calculations: Entrapment efficiency (%) = (weight of encapsulated drug / weight of drug added) × 100%, Loading capacity (%) = (weight of encapsulated drug / weight of nanoparticles) × 100%.

The release kinetics of 17-DMAG from PLGA-NHS were investigated using a cellulose dialysis bag (Molecular Weight: 3,000; Sigma, USA). 100 mg of PLGA-NHS loaded with 10 mg of 17-DMAG were dispersed in 10 mL of PBS buffer (pH 7.4) and stirred at room temperature. Aliquots were sampled at predetermined time intervals (0, 1, 2, 4, 8, 12, 24, 36, 48, 60, and 72 h), and were analyzed using HPLC.

### Purification of recombinant anti-CD19 scFv protein

The recombinant vector pMCSG7-His-anti-murine CD19 scFv was transformed into the protein expression strain Escherichia coli BL21 (DE3) pLysS 39. The bacteria cells were cultured overnight at 37°C and 180 rpm in a constant temperature shaker using complete Terrific Broth (TB) liquid medium supplemented with 100 μg/mL carbenicillin. The culture was then scaled up by a factor of 1:50, and protein expression was induced with 0.5 mM IPTG when the optical density (OD 600) reached 0.6 to 0.8. This was followed by incubation for 16 h at 16°C and 180 rpm. Bacterial cells were harvested by centrifugation at 4°C and 10,000 x g for 20 min and resuspended in 20 mM Tris-HCl buffer. Following ultrasonication, the supernatant was collected and applied to a His-affinity nickel chromatography column (Sigma, USA), followed by mixing for 2 h at 4°C. The column was washed with 10 mM imidazole Tris-HCl, and the anti-CD19 scFv protein (hereafter referred to as CD19 scFv) was eluted with 100 mM imidazole Tris-HCl. The sample was then ultrafiltered using an Amicon® Ultra centrifugal filter (Millipore, USA) and underwent buffer exchange with PBS (pH 7.4). Finally, the CD19 scFv was filtered through a 0.45 μm filter, endotoxin was removed, and the preparation was stored at 4°C. The spatial structure prediction of CD19 scFv was performed using the Phyre^2^ (https://www.sbg.bio.ic.ac.uk/phyre2).

### CD19 scFv targeting *in vitro* and *in vivo*

To evaluate the targeting capability of CD19 scFv, we performed protein binding assays both *in vitro* and *in vivo*. For the *in vitro* assay, 1 × 10^5^ splenocytes from healthy C57BL/6 mice were incubated with biotinylated CD19 scFv. The cells were subsequently stained with PE-streptavidin and APC-conjugated anti-mouse B220 antibody before being analyzed by flow cytometry. For the *in vivo* assay, healthy C57BL/6 mice were administered an intravenous (i.v.) injection of biotinylated CD19 scFv. Peripheral blood samples were collected 10 min post-injection, and a total of 1 × 10^5^ nucleated cells were stained with PE-streptavidin and APC-conjugated anti-mouse B220 antibody. Flow cytometric analysis was performed using a CytoFLEX system (Beckman Coulter, Inc.), and data were analyzed with Kaluza Analysis Software 2.1 (Beckman Coulter, Inc.).

### Cell lines and mice

The murine A70.2 pre-B cell line 40, Raw 264.7 macrophage cell line, EL4 T lymphoma cell line, NIH/3T3 fibroblast cell line, BCR-ABL1^+^ B-ALL cell line 23 and A20 lymphomas cell line were cultured at 37°C in a humidified atmosphere containing 5% CO_2_. The RPMI 1640, DMEM and IMDM media were supplemented with 10% heat-inactivated fetal bovine serum, 100 U/mL penicillin, and 100 U/mL streptomycin (HyClone^TM^, USA).

8-week-old C57BL/6, BALB/c and OT-1 transgenic mice were housed in the specific pathogen-free (SPF) conditions under the Xi'an Jiaotong University Division of Laboratory Animal Research. Experimental procedures were accorded by the Institutional Animal Care and Use Committee of Xi'an Jiaotong University.

### Biocompatibility and biosafety evaluation

Cells were cultured in 96-well plates at a density of 1 × 10^5^ cells/mL (100 µL/well), with the outer well of the plates filled with PBS to minimize edge effects. PLGA-NHS nanoparticles were added to the wells at various concentrations (10, 50, 100, 200, 400, and 600 µg/mL), with three replicates for each concentration. After incubation for 24 h or 48 h, 10 µL of CCK-8 reagent (5 mg/mL) was added to each well and incubated for an additional 4 h. Absorbance was then measured at 450 nm using a microplate reader (Thermo Fisher Scientific, USA).

To assess the *in vivo* biocompatibility, healthy C57BL/6 mice were intravenously administered either PLGA-NHS nanoparticles (15 mg in PBS) or PBS buffer (as a vehicle control) daily for seven consecutive days. Blood samples were collected for routine biochemical and hematological analyses using VetScan VS2 and VetScan HM5 analyzers (Abaxis), respectively. Vital organs, including the heart, liver, lung, and kidney, were harvested and processed for hematoxylin and eosin (H&E) staining. The histological sections from PLGA-NHS-treated mice were compared with those from the vehicle-treated group to assess any potential organ toxicity.

### Preparation and characterization of CD19 scFv conjugated to PLGA-NHS

Lyophilized PLGA-NHS was dissolved in 50 mL of PBS. A freshly prepared CD19 scFv protein solution was then added at a 1:1 molar ratio, and the mixture was gently stirred overnight at 4 °C. Following incubation, the mixture was centrifuged at 15,000 rpm for 15 min at 4 °C. The supernatant was discarded, and the precipitate was washed and resuspended in PBS. This centrifugation and washing step were repeated three times to remove any unbound proteins. The resulting pellet was flash-frozen in liquid nitrogen and lyophilized.

The conjugation of CD19 scFv to PLGA-NHS, hereafter referred to as CD19@NP, was validated through Fourier-transform infrared (FTIR) spectroscopy (Bruker VERTEX70, Germany). Additional characterization of CD19@NP, including assessments of its biocompatibility and biosafety, was performed following the protocols previously established for PLGA-NHS.

### The distribution and specific targeting ability of CD19@NP *in vivo*

Normal C57BL/6 mice were intravenously injected with either DiR-loaded PLGA-NHS nanoparticles or DiR-loaded CD19@NP. Fluorescence imaging was carried on using an *in vivo* imaging system (IVIS, PerkinElmer, USA) at predetermined time points. Subsequently, the mice were euthanized, and their major organs (heart, liver, spleen, lung, kidney, and bone marrow) were harvested for *ex vivo* imaging using the same IVIS system.

The targeting ability of CD19@NP were assessed following the same protocols used for CD19 scFv. A competitive binding assay was also performed to examine specificity of CD19@NP targeting. First, 1 × 10^5^ A70.2 pre-B cells were preincubated with either the irrelevant protein bovine serum albumin (BSA) or free CD19 scFv for 30 min, then washed twice with cold PBS to remove unbound proteins. The cells were subsequently incubated with biotinylated CD19@NP, followed by two additional PBS washes to eliminate any unbound nanoparticles. In parallel, another set of A70.2 B cells was incubated with vehicle control, free CD19 scFv, biotinylated CD19 scFv, or biotinylated CD19@NP. All samples were then analyzed by flow cytometry.

### Phagocytosis assay of nanoparticles

A total of 1 × 10^5^ RAW264.7 macrophages cells were incubated with 400 µg/mL of DiL-loaded PLGA-NHS or DiL-loaded CD19@NP at various time points (0, 1, 2, 4, 8, 12, and 24 h). Phagocytosis was quantified by flow cytometry as the percentage of DiL⁺ RAW264.7 cells. Similarly, 1 × 10^5^ splenocytes from C57BL/6 mice were incubated under the same conditions and then stained with PC7 anti-mouse CD11b antibodies. Phagocytosis in this group was measured as the percentage of DiL⁺ CD11b⁺ myeloid cells.

### Treatment strategies in BCR-ABL1^+^ B-ALL and A20 lymphomas mouse model

The murine BCR-ABL1/GFP^+^ leukemia cell line has been previously described 23. A total of 1 × 10^4^ leukemia cells were injected into non-irradiated, immunocompetent C57BL/6 hosts to establish the BCR-ABL1^+^ B-ALL mouse model. Treatment was initiated when GFP^+^ leukemia cells in peripheral blood reached 1%. The treatment regimens were as follows: Imatinib (100 mg/kg) or ponatinib (10 mg/kg) was administered once daily via oral gavage. Vehicle (0.5% CMC-Na) was administered once daily via oral gavage as a control. 17-DMAG (10 mg/kg) was administered intraperitoneally every other day. CD19@NP/17-DMAG (10 mg/kg) was administered via tail vein injection once every three days. All treatments were continued for two weeks.

To establish the A20 lymphoma subcutaneous tumor model, 5 × 10^6^ A20 lymphoma cells were subcutaneously injected into immunocompetent BALB/c mice. Treatment was initiated when the tumor volume reached 100 to 200 mm^3^. The treatment regimens were as follows: 17-DMAG (10 mg/kg) was administered intraperitoneally every other day. CD19@NP/17-DMAG (10 mg/kg) was administered intravenously once every three days. Both treatments were continued for two weeks. Tumor size was measured every two days using vernier calipers. Tumor volume was calculated using the ellipsoid volume formula: V = π/6 × L × W^2^ (L, the longest diameter of the tumor; W, the shortest diameter of the tumor).

### *In vivo* T-cell depletion assay

To deplete CD4^+^ and CD8^+^ T cells, mice received intraperitoneal injections of 150 µg of depleting antibodies one day before treatment and every three days thereafter. For CD4^+^ T-cell depletion, *InVivo*MAb anti-mouse CD4 (Bio X Cell, clone: YTS 191) was used, while *InVivo*MAb anti-mouse CD8α (Bio X Cell, clone: 53-6.7) was employed for CD8^+^ T-cell depletion. The effectiveness of the depletion was verified through flow cytometry analysis using anti-mouse CD4 and anti-mouse CD8α antibodies.

### T-cell *in vitro* activation and proliferation

Splenocytes were isolated from 8- to 10-week-old OT-1 mice. CD8⁺ T cells were purified using the Mouse CD8⁺ T Cell Isolation Kit (Selleck, B90011) via negative selection, following the manufacturer's instructions. Purified CD8⁺ T cells were stimulated with plate-bound anti-CD3ε (5 μg/mL) and anti-CD28 (1 μg/mL) antibodies for 24 h. Subsequently, 3 × 10⁵ activated CD8⁺ T cells were cocultured with 1.5 × 10⁵ BCR-ABL1⁺ B-ALL cells that had been pretreated *in vitro* for 24 h with PBS, imatinib plus 17-DMAG, or imatinib plus CD19@NP/17-DMAG in the presence of 1 µg/mL OVA peptide (SIINFEKL). Cocultures were maintained for either 24 or 72 h to assess T cell activation and proliferation, respectively.

### *B2M* knockout by CRISPR/Cas9

Guide RNA (gRNA) oligos targeting the mouse *B2m* gene were designed using online software (http://crispr.mit.edu/). Four gRNA oligos were synthesized and annealed to produce two DNA fragments (1F 5'-caccgAGTATACTCACGCCACCCAC-3', 1B 5'-aaacGTGGGTGGCGTGAGTATACTc-3'; 2F 5'-caccgCGTATGTATCAGTCTCAGTG-3', 2B 5'-aaacCACTGAGACTGATACATACGc-3'). These fragments were then cloned into the LentiCRISPRv2 vector. Viral supernatants from the newly constructed LentiCRISPRv2 plasmids were used to infect BCR-ABL1^+^ B-ALL cells. Transduced cells were selected using 0.5 µg/mL puromycin. After approximately a week, drug-resistant cells emerged. The knockout efficiency was evaluated via flow cytometry using anti-mouse MHC-I antibodies.

### Re-challenge experiments

C57BL/6 mice were initially challenged with 1 × 10^4^ BCR-ABL1^+^ B-cells via tail vein injection and treated as described previously. The absence of leukemia in the peripheral blood was confirmed using flow cytometry. Six weeks after the start of treatment, mice with no detectable leukemia were subsequently re-challenged with a 10-fold greater number of leukemia cells (1 × 10^5^) via tail vein injection. Naïve C57BL/6 mice were also challenged with the same number of leukemia cells to serve as controls.

Immune cell populations were analyzed by flow cytometry on day 9 post-re-challenge.

### RNA sequencing and data analysis

Total RNA was extracted from GFP^+^ splenocytes of treatment mice (three samples per group) using TRIzol (Takara Bio), followed by purification for library preparation and sequencing on an Illumina HiSeq™ platform. Raw single-strand sequencing data were aligned using the Salmon package and mapped to the GRCm38 reference genome. Transcripts per million (TPM) values were calculated for each gene. Differential expression analysis was carried out with the DESeq2 R package, considering genes as significantly differentially expressed when the fold change exceeded 2 and the adjusted *p* value (*q* value) was below 0.05. Kyoto Encyclopedia of Genes and Genomes (KEGG) pathway analysis for differentially expressed genes (DEGs) was performed using the clusterProfiler R package.

### Statistical analysis

Statistical analyses were performed using GraphPad Prism software. All results are expressed as mean ± standard error of the mean (SEM). A *p* value < 0.05 was considered statistically significant, with significance levels indicated as follows: *P < 0.05, **P < 0.01, ***P < 0.001, and ****P < 0.0001. Survival curves were compared using the Log-rank (Mantel-Cox) test. For comparisons between two groups, Student's t-test was used to assess statistical significance. Data sets containing more than two groups were analyzed using two-way ANOVA.

## Results

### Preparation and characterization of biocompatible complex nanoparticles composed of PLGA and PLA-PEG-NHS

**Figure 1:**
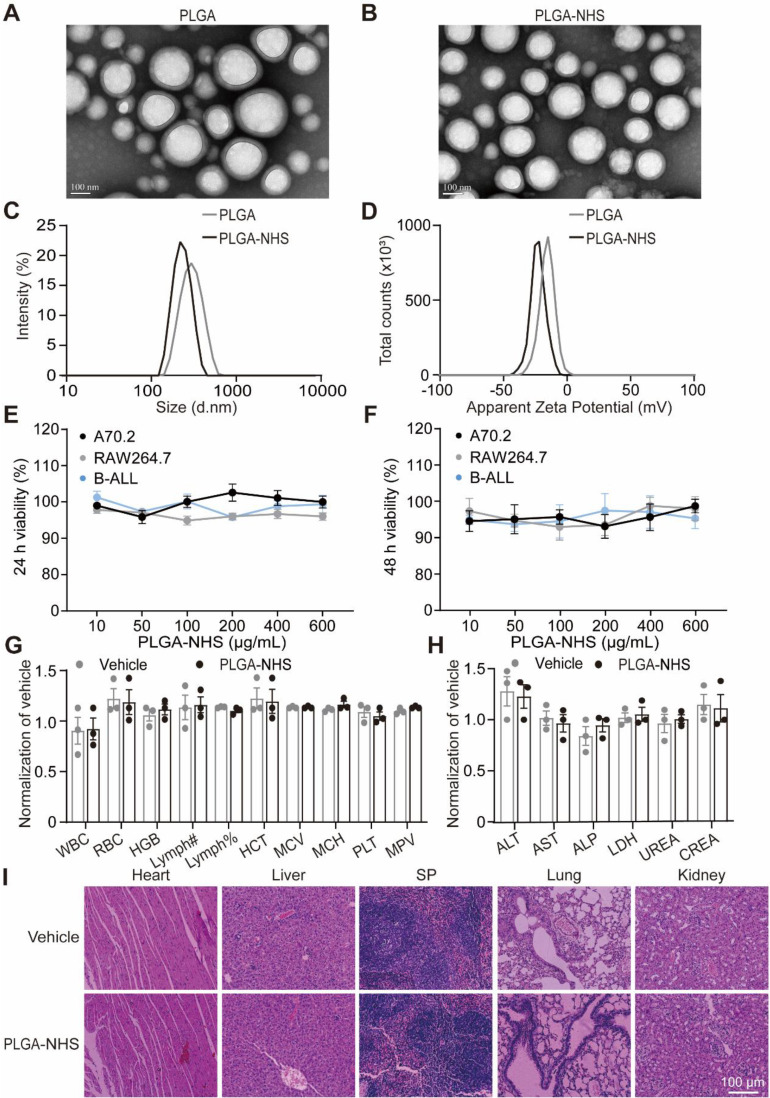
Characterization and biocompatibility of PLGA and PLGA-NHS nanoparticles. (A-B) TEM images show the morphology of PLGA (A) and PLGA-NHS (B) nanoparticles. (C-D) DLS measurements reveal the size distribution (C) and zeta potential (D) of PLGA and PLGA-NHS nanoparticles. (E-F) *In vitro* cytotoxicity assessment of PLGA-NHS nanoparticles on A70.2 pre-B cells, Raw 264.7 macrophage and BCR-ABL1^+^ B-ALL cells. The cells were incubated with PLGA-NHS for 24 h (E) and 48 h (F), followed by evaluation using the CCK-8 assay. Data represent the average of 3 independent experiments with error bars indicating the SEM. (G-H) Blood samples were collected from healthy C57BL/6 mice (n = 3) and analyzed for hematological (G) and biochemical (H) parameters. WBC (total white blood cells), RBC (red blood cells), HGB (hemoglobin), Lymph# (absolute lymphocyte count), Lymph% (lymphocyte percentage), HCT (hematocrit), MCV (mean corpuscular volume), MCH (mean corpuscular hemoglobin), PLT (platelets), and MPV (mean platelet volume). ALT (alanine aminotransferase), AST (aspartate aminotransferase), ALP (alkaline phosphatase), LDH (lactate dehydrogenase), UREA (urea), and CREA (creatinine). (I) H&E staining of heart, liver, spleen, lung, and kidney tissues from healthy C57BL/6 mice treated daily for 7 days with either PLGA-NHS or PBS (as a control), followed by one month of observation. Scale bar represents 100 μm.

To develop targeted nano-delivery systems, we synthesized two types of nanoparticles: PLGA and PLGA modified with PLA-PEG-NHS (PLGA-NHS) using a single-step functionalization technique 38. TEM imaging confirmed that both nanoparticles exhibited well-defined, spherical shapes with uniform sizes (Figure 1A-B). Dynamic light scattering analysis revealed that PLGA nanoparticles had an average diameter of 287.2 ± 0.931 nm and a zeta potential of -15.9 ± 5.54 mV, while PLGA-NHS nanoparticles were smaller, with an average diameter of 220.7 ± 0.964 nm and a more negative zeta potential of -23.2 ± 6.41 mV (Figure 1C-D). Both types demonstrated narrow size distributions, with polydispersity indexes of 0.104 for PLGA and 0.040 for PLGA-NHS (Table 1). High-performance liquid chromatography (HPLC) analysis showed that loading 10 mg of 17-DMAG into PLGA-NHS resulted in a drug loading efficiency of 10.8 ± 0.98% and an encapsulation efficiency of 65 ± 0.07% (Figure S1A, Table 2). The release profile indicated that approximately 55% of the HSP90 inhibitor 17-DMAG was released within the first 24 h, with about 80% released over 72 h (Figure S1B). Collectively, PLGA-NHS demonstrates decent drug-loading capacity, and NHS modification enhances the compactness and stability of the nanoparticles.

To test the biocompatibility of PLGA-NHS *in vitro*, we treated murine A70.2 pre-B cells, RAW 264.7 macrophages, and primary BCR-ABL1^+^ B-ALL cells with varying concentrations of PLGA-NHS for 24 h and 48 h. The treatment did not affect cell viability (Figure 1E-F). *In vivo* experiments were undertaken by administering 15 mg of PLGA-NHS daily to healthy C57BL/6J mice for a week. The treated mice showed no significant changes in body weight (Figure S1C), organ appearance (Figure S1D), blood parameters (Figure 1G), biochemical levels (Figure 1H), or tissue structure (Figure 1I) compared to the saline-treated control group. These results indicate that PLGA-NHS nanoparticles are safe for use as a drug delivery system.

### Preparation and characterization of CD19@NP

CD19, a pan B-cell antigen, serves as an excellent candidate target for therapies against B-lineage leukemia and lymphoma. We engineered a recombinant mouse anti-CD19 single-chain variable fragment (scFv) that specifically binds to the extracellular domain of the CD19 protein, as described previously (Figure S2A) 39. The CD19 scFv, with a molecular weight of approximately 27.5 kDa, was purified to 97% purity using Ni-NTA affinity chromatography (Figure S2B). To evaluate its specificity, binding assays with splenic B220⁺ B cells demonstrated that 93% were bound by CD19 scFv compared to 0.6% by the vehicle control *in vitro* (Figure S2C). Similarly, approximately 98% of splenic B220⁺ B cells were bound by CD19 scFv compared to 0.2% by the vehicle control *in vivo* (Figure S2D), confirming that the CD19 scFv generated in this study specifically targets mouse CD19 on the B-cell surface.

**Figure 2:**
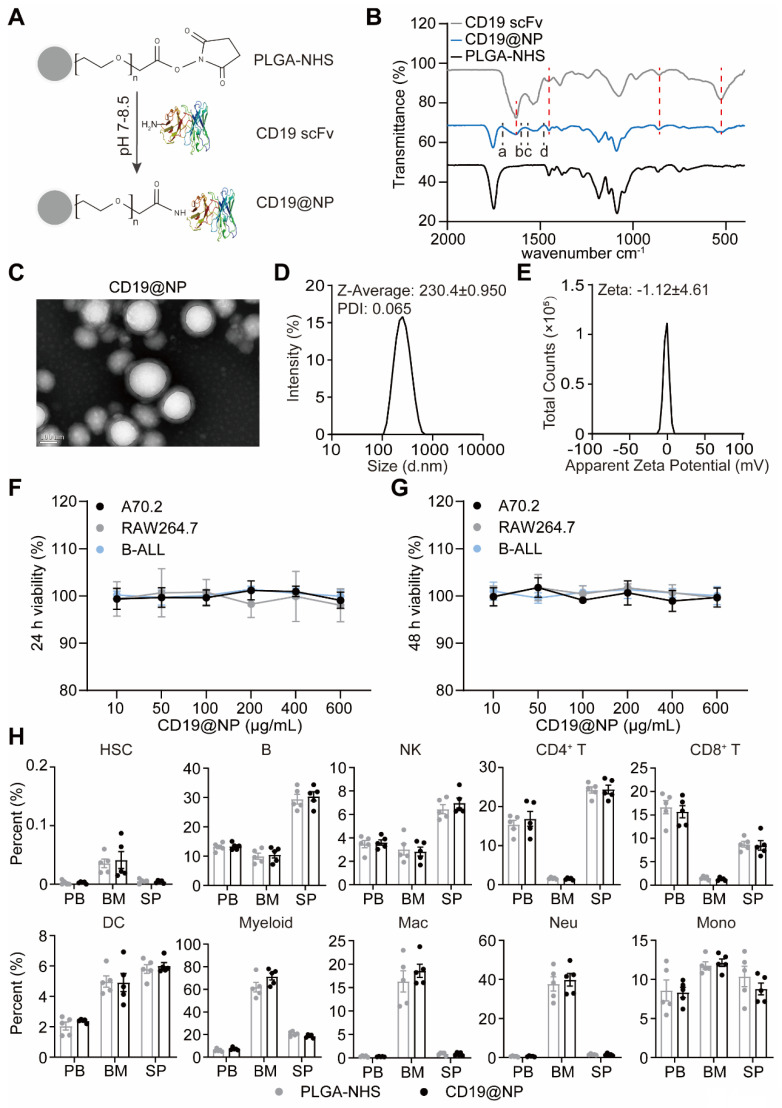
Characterization and biocompatibility of CD19@NP. (A) Schematic representation of the CD19@NP synthesis process. (B) FTIR spectra of CD19 scFv, CD19@NP and PLGA-NHS. The red dashed lines represent the overlapping infrared absorption peaks of CD19@NP and CD19 scFv, while the black dashed lines (a-d) denote the characteristic peaks of the newly formed amide bonds in CD19@NP. (C-E) Characterization of CD19@NP: TEM images (C), size distribution (D) and zeta potential (E). (F-G) *In vitro* cytotoxicity assessment of CD19@NP on A70.2, Raw 264.7 and BCR-ABL1^+^ B-ALL cells. The cells were incubated with PLGA-NHS for 24 h (F) and 48 h (G), followed by evaluation using the CCK-8 assay. Data represent the average of 3 independent experiments with error bars indicating the SEM. (H) The *in vivo* biosafety of CD19@NP was assessed through flow cytometry analysis of the indicated cell populations following *in vivo* injection of PLGA-NHS and CD19@NP into C57BL/6 mice (n = 5). The analyzed cell populations included HSCs (hematopoietic stem cells, Lineage⁻ c-kit⁺ sca-1⁺), B (CD19⁺ cells), NK (natural killer cells, CD3⁻ NK1.1⁺), CD4⁺ T (CD3⁺ CD4⁺ cells), CD8⁺ T (CD3⁺ CD8⁺ cells), DC (dendritic cells, CD11c⁺), myeloid (CD11b⁺ cells), Mac (macrophages, CD11b⁺ F4/80⁺), Neu (neutrophils, CD11b⁺ Ly6G⁺), and Mono (monocytes, CD11b⁺ Ly6C⁺ Ly6G⁻) in peripheral blood (PB), bone marrow (BM), and spleen (SP) after treatment with PLGA-NHS (gray dots) and CD19@NP (black dots). Data represent the average of 5 mice, with error bars indicating the SEM.

Building on this specificity, previous studies have demonstrated that the NHS groups on PLGA-NHS nanoparticles exhibit high reactivity with primary amines of proteins, forming stable amide bonds that effectively anchor proteins onto the nanoparticle surface. Using Phyre^2^ for 3D structure prediction, we identified primary amine sites on the CD19 scFv, including N-terminal amines and lysine side chains (Figure S2E). To harness this potential, we conjugated CD19 scFv to PLGA-NHS nanoparticles (CD19@NP) at a 1:1 molar ratio (Figure 2A). Fourier Transform Infrared Spectroscopy (FTIR) analysis identified two characteristic infrared absorption peaks corresponding to the specific amide bonds formed during the synthesis of CD19@NP: the Amide I (1640-1660 cm^-1^) and Amide II (~1540 cm^-1^) bands (Figure 2B). Further validation using Coomassie Brilliant Blue staining and Western blotting revealed that CD19@NP exhibited the same molecular weight and His-tag-specific bands as free CD19 scFv, verifying the presence of CD19 scFv on the nanoparticles (Figure S3A). All these results confirm the successful preparation of CD19@NP.

To further evaluate CD19@NP, we next characterized its physical properties and biocompatibility. CD19@NP retained the original PLGA-NHS structure, indicating minimal physical changes following surface decoration (Figure 2C). Successful coating with anti-CD19 scFv was confirmed by an increase in particle diameter, accompanied by a zeta potential shift to -1.12 ± 4.61 mV (Figure 2D-E), suggesting a slight reduction in colloidal stability. After exposure to varying concentrations of PLGA-NHS or CD19@NP for 24 h or 48 h *in vitro*, no significant effects on the viability of various cell types were observed including murine A70.2 pre-B cells, RAW 264.7 macrophages, primary BCR-ABL1⁺ B-ALL cells, A20 B lymphoma cells, EL4 T cells, and NIH/3T3 fibroblast cells (Figure 2F-G, Figure S3B-C). *In vivo*, 15 mg of either PLGA-NHS or CD19@NP was administered via tail vein injection into groups of five healthy C57BL/6 mice, followed by flow cytometric analysis of peripheral blood, bone marrow, and spleen samples after 72 h. No significant differences were detected between the two groups in the proportions of hematopoietic stem cells (HSCs), B cells, NK cells, CD4⁺ and CD8⁺ T cells, dendritic cells, or myeloid cells, including macrophages, neutrophils, and monocytes (Figure 2H). These results demonstrate that CD19@NP is biocompatible and does not disrupt immune cell populations either *in vitro* or *in vivo*.

### The specific targeting capability and prolonged *in vivo* biodistribution of CD19@NP

**Figure 3:**
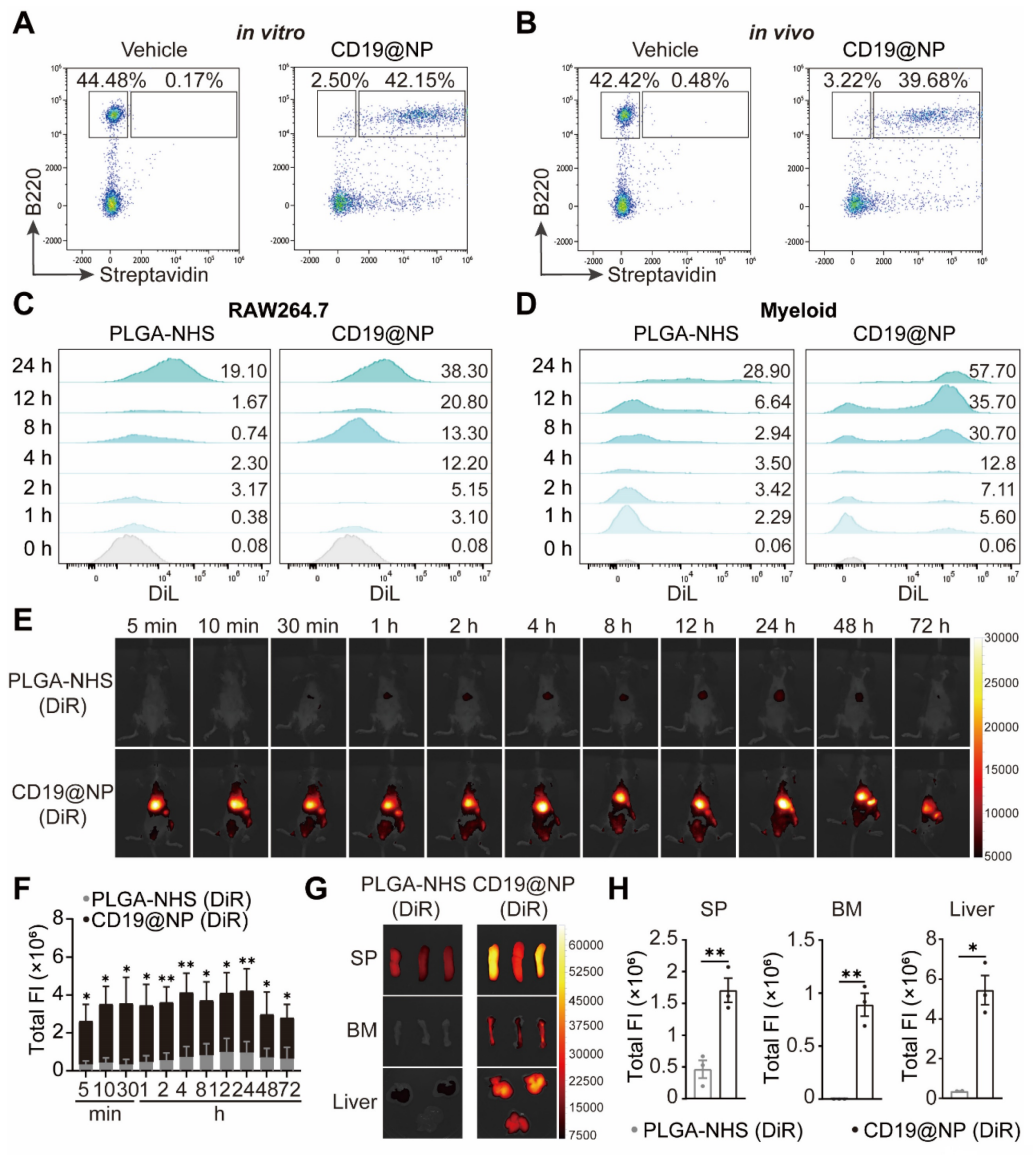
The specific targeting ability and *in vivo* biodistribution of CD19@NP. (A-B) *In vitro* and *in vivo* targeting of CD19@NP. (A) The targeting ability *in vitro* of CD19@NP was examined by incubating splenocytes with biotin-labeled CD19@NP, with free CD19 scFv serving as a negative control. After incubation, the cells were stained with anti-mouse B220 antibody and streptavidin and analyzed by flow cytometry. (B) The targeting ability *in vivo* of CD19@NP was assessed by intravenously injecting mice with biotin-labeled CD19@NP, with free CD19 scFv serving as a negative control. Peripheral blood samples were collected 10 min post-injection, and the cells were stained with anti-mouse B220 antibody and streptavidin, followed by analysis using flow cytometry. (C) Phagocytosis of DiL-loaded PLGA-NHS and DiL-loaded CD19@NP by RAW264.7 macrophages was assessed at the indicated time points (0, 1, 2, 4, 8, 12, and 24 h) using flow cytometry. The values above each peak indicate the percentage of phagocytosed cells at the corresponding time point for PLGA-NHS (left) and CD19@NP (right). (D) Phagocytosis of DiL-loaded PLGA-NHS and DiL-loaded CD19@NP by mouse primary splenic myeloid cells from C57BL/6 mice was analyzed under the same conditions and time points, with flow cytometry used to determine the percentage of phagocytosed cells for PLGA-NHS (left) and CD19@NP (right). (E-F) DiR-loaded PLGA-NHS and CD19@NP were intravenously injected into C57BL/6 mice (n = 3). Images were obtained at the indicated time points post-injection using an *in vivo* imaging system (IVIS) (E). Total fluorescence intensity (FI) was quantified (F). (G-H) Organ biodistribution was investigated 72 h post-injection using IVIS (G). Total FI was quantified (H). Unless otherwise stated, results are presented as the means ± SEM: *P < 0.05, **P < 0.01, ***P < 0.001 and ****P < 0.0001. Stars indicate a significant *p* value as calculated by the relevant statistical test.

To confirm the targeting capability of CD19@NP, we performed binding assays using splenic B220⁺ B cells. *In vitro*, 94% of B220⁺ B cells were bound by CD19@NP, compared to only 0.3% bound by the vehicle control (Figure 3A). *In vivo*, 93% of splenic B220⁺ B cells were bound by CD19@NP, whereas only 0.1% were bound by the vehicle control (Figure 3B). To further investigate binding dynamics, a competitive binding assay was undertook using the A70.2 B-cell line. Cells were incubated with the vehicle control, free CD19 scFv, biotinylated CD19 scFv, or biotinylated CD19@NP. For blocking experiments, A70.2 cells were pre-incubated with either BSA or CD19 scFv prior to exposure to biotinylated CD19@NP. Flow cytometry analysis revealed that biotinylated CD19 scFv and biotinylated CD19@NP bound to A70.2 cells with efficiencies of 96% and 93%, respectively. Pre-incubation with BSA had minimal effect on binding, with 94% of cells still bound by CD19@NP, while pre-incubation with CD19 scFv significantly reduced binding to 4.7% (Figure S3D), further supporting that the specificity of CD19@NP targeting.

To explore whether CD19 scFv modification influenced the phagocytosis of PLGA-NHS, we assessed the uptake of DiL-loaded PLGA-NHS and CD19@NP by RAW264.7 macrophages and primary splenic myeloid cells derived from wild-type C57BL/6 mice *in vitro*. Flow cytometry revealed a significant increase in the phagocytosis of CD19@NP compared to PLGA-NHS (Figure 3C-D). Given that this increase could stem from non-specific interactions rather than specific targeting, we performed additional binding assays with CD19@NP for primary splenic myeloid cells. These assays demonstrated that CD19@NP did not exhibit specific binding to myeloid cells (Figure S3E), suggesting that the observed enhancement in phagocytosis is likely non-specific.

Finally, to evaluate the biodistribution of CD19@NP, we utilized an *In Vivo* Imaging System (IVIS) to track DiR-labeled PLGA-NHS and CD19@NP following intravenous injection into C57BL/6 mice. Although CD19@NP demonstrated susceptibility to phagocytosis by myeloid cells *in vitro*, *in vivo* imaging revealed rapid localization to target organs, including the spleen, bone marrow, and liver, within 5 min of injection (Figure 3E-F). Significantly higher accumulation of CD19@NP was observed in these organs compared to PLGA-NHS at 72 h post-injection (Figure 3E-F). *Ex vivo* imaging of dissected organs at 72 h confirmed markedly greater fluorescence intensity in the spleen, bone marrow, and liver for CD19@NP compared to PLGA-NHS, with minimal fluorescence detected in the heart and lung (Figure 3G-H, Figure S3F). Despite this enhanced uptake, CD19@NP retained its specific targeting ability and demonstrated an extended metabolic half-life *in vivo*, underscoring its potential as a promising candidate for targeted drug delivery applications.

### CD19@NP/17-DMAG plus imatinib reduce disease burden and improve survival in BCR-ABL1^+^ B-ALL

**Figure 4:**
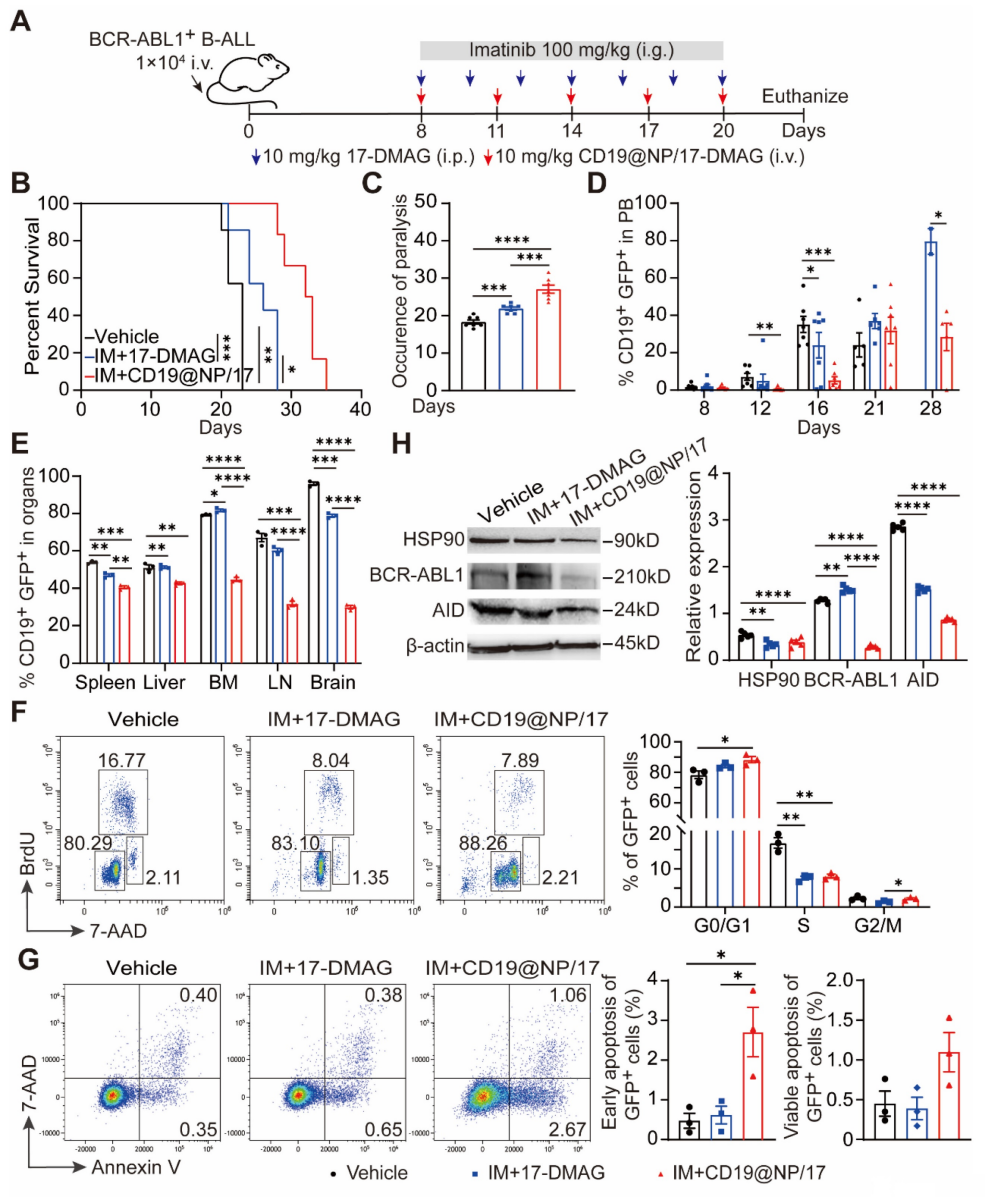
Reduction of leukemic burden in BCR-ABL1^+^ B-ALL mice treated with CD19@NP/17-DMAG. (A) Overview of the indicated treatment options for BCR-ABL1^+^ B-ALL mice. (B) Kaplan-Meier survival curves of BCR-ABL1^+^ B-ALL mice treated with vehicle, imatinib (IM) combined with 17-DMAG, or CD19@NP/17-DMAG (n = 7). (C) Time to leukemia cell infiltration in the nervous system for each treatment group above. (D) Percentage of CD19^+^ GFP^+^ cells in peripheral blood of BCR-ABL1^+^ B-ALL mice treated with vehicle, IM combined with 17-DMAG, or CD19@NP/17-DMAG at indicated timepoints (n = 7). (E) Percentage of CD19^+^ GFP^+^ cells in spleen (SP), liver, bone marrow (BM), lymph nodes (LN), and brain of moribund mice after the indicated treatments. (F) BrdU cell proliferation assay on splenic GFP^+^ cells derived from BCR-ABL1^+^ B-ALL mice (n = 3) following the indicated treatments. (G) Apoptosis assay on GFP^+^ cells from spleen in the indicated treatment groups (n = 3). 7-AAD^-^ Annexin V^+^ and 7-AAD^+^ Annexin V^+^ cells represent early and late apoptosis cells, respectively. (H) Western blotting analysis of HSP90, BCR-ABL1 and AID in BCR-ABL1^+^ B-ALL leukemic cells from spleen of moribund deceased mice following the treatments. The same blot was reprobed with an anti-β-actin antibody to assess protein loading. Unless otherwise stated, results are presented as the means ± SEM: *P < 0.05, **P < 0.01, ***P < 0.001 and ****P < 0.0001. Stars indicate a significant *p* value as calculated by the relevant statistical test.

Our previous research showed that combining 17-DMAG with imatinib extended survival in BCR-ABL1^+^ B-ALL mouse models 23. To examine whether CD19@NP/17-DMAG with imatinib is more effective than 17-DMAG plus imatinib alone (Figure 4A), we employed a previously described murine BCR-ABL1⁺ B-ALL model 23, which mirrors patient-derived Ph⁺ B-ALL through the rapid proliferation of CD19⁺ GFP⁺ leukemic cells in the bone marrow and secondary lymphoid organs (Figure S4A-C). Notably, the combination of CD19@NP/17-DMAG and imatinib significantly prolonged survival by 10 days compared to vehicle controls, and by 7 days compared to 17-DMAG plus imatinib (Figure 4B). In addition, this regimen more effectively limited leukemic cell spread to the nervous system and other organs relative to imatinib plus 17-DMAG (Figure 4C-E, Figure S4D-E).

Cell cycle analysis revealed that CD19@NP/17-DMAG treatment decreased the proportion of GFP⁺ leukemic cells in the S and G2/M phases while increasing those in G0/G1 (Figure 4F). Moreover, this combination therapy significantly elevated both early and late apoptotic cell populations compared to controls (Figure 4G). Critically, the expression of key HSP90 client proteins, including BCR-ABL1 and activation-induced cytidine deaminase (AID), was markedly lower in the group treated with CD19@NP/17-DMAG and imatinib. (Figure 4H). Collectively, these results show that this combined therapy substantially enhances efficacy by inducing apoptosis, reducing tumor burden, and delaying disease progression.

### Transcriptional reprogramming in BCR-ABL1^+^ B-ALL cells by CD19@NP/17-DMAG combined with imatinib

**Figure 5:**
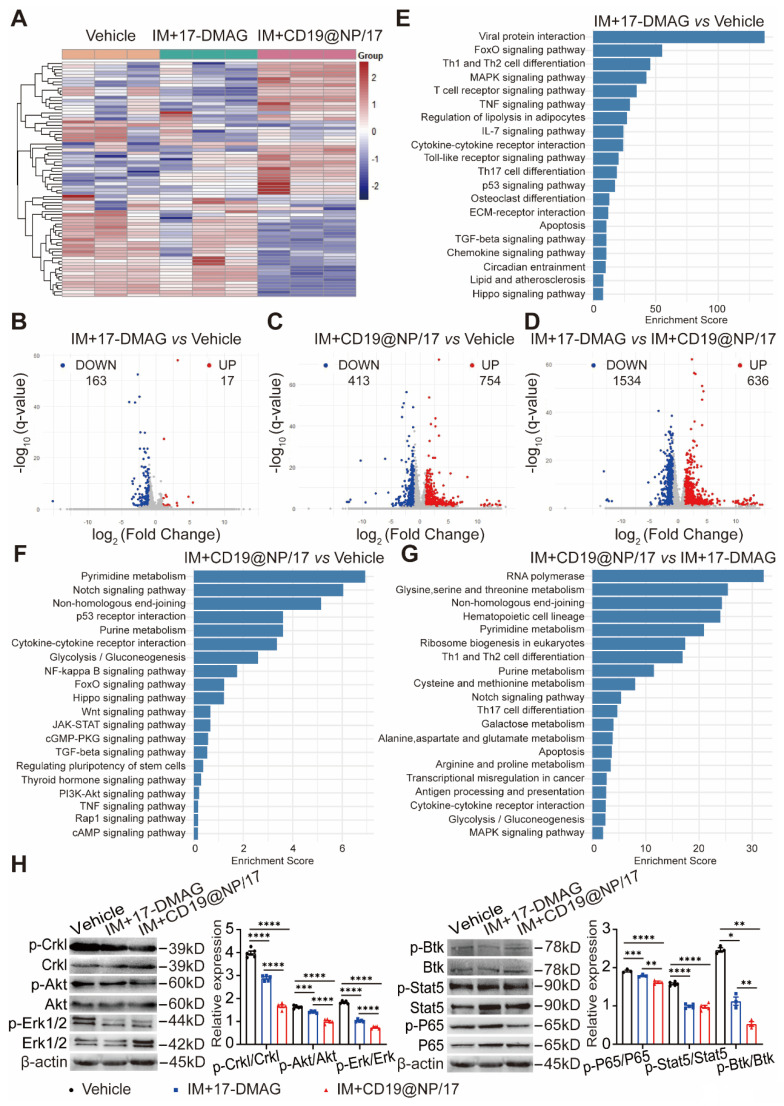
Pathway enrichment analysis and validation of RNA-seq in BCR-ABL1^+^ B-ALL. (A) Heatmap showing the hierarchical clustering of differentially expressed genes (DEGs) between the three groups: Vehicle, IM + 17-DMAG, and IM + CD19@NP/17-DMAG (n = 3). (B-D) Volcano plots displaying DEGs for the comparisons of IM + 17-DMAG *vs* Vehicle (B), IM + CD19@NP/17-DMAG *vs* Vehicle (C) and IM + 17-DMAG* vs* IM + CD19@NP/17-DMAG (D). DEGs were identified by fold change > 2 and *q* value < 0.05. Upregulated genes are marked in red and downregulated genes in green. The numbers of significantly up- and downregulated genes are indicated. (E-G) KEGG pathway enrichment analysis for DEGs in the following comparisons: IM + 17-DMAG* vs* Vehicle (E), IM + CD19@NP/17-DMAG *vs* Vehicle (F), and IM + 17-DMAG* vs* IM + CD19@NP/17-DMAG (G). Pathways are ranked by enrichment score, with key signaling pathways related to immune responses, metabolism, and cell signaling highlighted in each comparison. (H) Western blotting analysis of the levels of p-CrkL, p-Akt, p-Erk, p-p65, p-Stat5 and p-Btk compared to their corresponding total proteins in samples subjected to the indicated treatments (n = 3-5). Unless otherwise stated, results are presented as the means ± SEM: *P < 0.05, **P < 0.01, ***P < 0.001 and ****P < 0.0001. Stars indicate a significant *p* value as calculated by the relevant statistical test.

To elucidate the molecular mechanisms underlying the enhanced therapeutic effects of combining CD19-directed 17-DMAG nanoparticles with imatinib compared to 17-DMAG plus imatinib, we isolated splenic GFP^+^ CD19^+^ cells from BCR-ABL1^+^ B-ALL mice after the indicated treatments (Figure 4A) and performed RNA-seq analysis. We identified differentially expressed genes (DEGs) using criteria of fold change > 2 and *q* value < 0.05. A heat map displayed the clustering of DEGs across all three treatment groups (Figure 5A). Principal component analysis (PCA) highlighted distinct global gene expression patterns, with PC1 and PC2 accounting for 19.126% and 33.782% of the variance, respectively (Figure S5A), indicating clear differences among the treatment groups.

A Venn diagram revealed overlapping and unique DEGs among the groups (Figure S5B), while volcano plots illustrated upregulated and downregulated DEGs in pairwise comparisons, highlighting significantly altered genes between the treatment groups (Figure 5B-D). KEGG pathway analysis showed that 17-DMAG plus imatinib primarily affected several cell proliferation-related pathways, such as FoxO, MAPK, p53, and Hippo signaling (Figure 5E). In contrast, CD19-targeted 17-DMAG delivery combined with imatinib impacted a broader range of pathways, including those related to cell differentiation, proliferation, and apoptosis, such as Notch, p53, NF-κB, FoxO, Wnt, JAK-STAT, cGMP-PKG, PI3K-Akt, cAMP, and MAPK signaling pathways (Figure 5F-G). Western blotting supported these findings, showing reduced phosphorylation of proliferation-related proteins (Crkl, Akt, Erk, Btk, Stat5, and NF-κB) (Figure 5H) and alterations in apoptosis regulators, with lower levels of anti-apoptotic proteins (Bcl-2, Bcl-xL, Mcl-1) and higher levels of pro-apoptotic proteins (Bad, Caspase-3, and Cytochrome c) (Figure S5C). Additionally, the targeted delivery of 17-DMAG affected various metabolic pathways, such as glycolysis/gluconeogenesis, pyrimidine, purine, galactose, and related amino acid metabolism (Figure 5F-G). The data imply that the nanoparticle-based delivery of 17-DMAG enhances leukemia cell apoptosis by upregulating pro-apoptotic genes and downregulating proliferation and anti-apoptotic genes, while also reducing glycolytic metabolism. This multifaceted effect ultimately improves survival outcomes in leukemic mice.

### CD19@NP/17-DMAG and imatinib treatment triggers T-cell immunity against BCR-ABL1^+^ B-ALL

**Figure 6:**
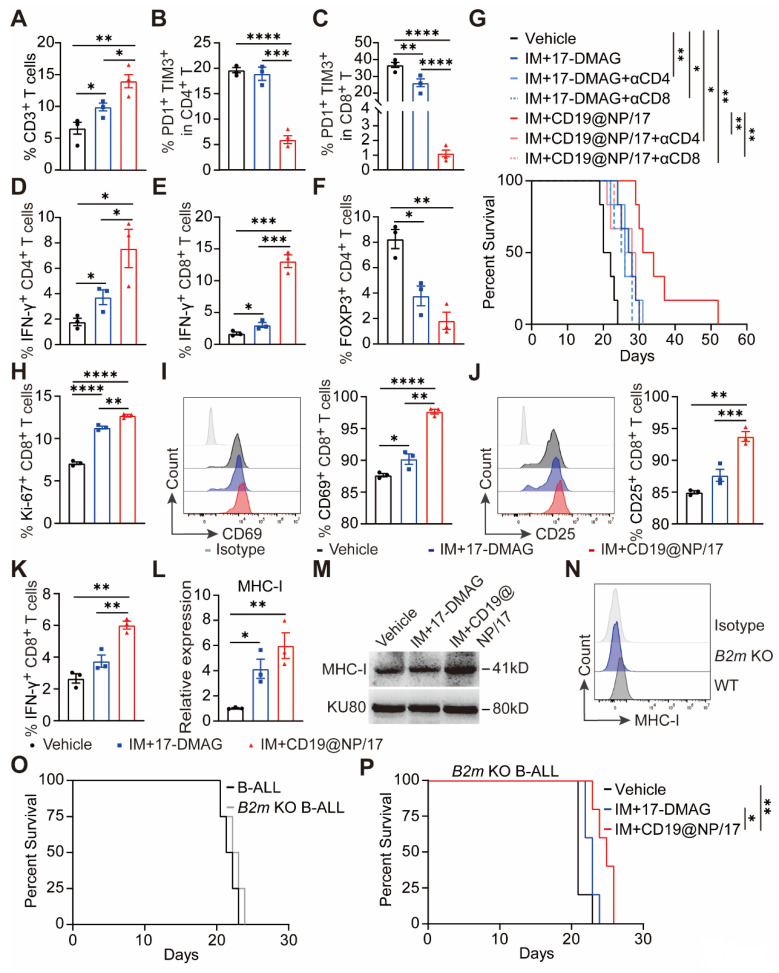
CD19@NP/17-DMAG enhances T-cell response and upregulates tumor-derived MHC-I in BCR-ABL1^+^ B-ALL. (A-F) Analysis of different T cell populations from spleen in BCR-ABL1^+^ B-ALL mice post one week of treatment. A bar graph illustrated the percentages of total CD3^+^ T (A), exhausted CD4^+^ T (B), exhausted CD8^+^ T (C), IFN-γ^+^ CD4^+^ T (D), IFN-γ^+^ CD8^+^ T (E) and regulatory cells (Treg) (F) in the spleen of BCR-ABL1^+^ B-ALL following the indicated treatment. (G) Kaplan-Meier survival curves of BCR-ABL1^+^ B-ALL mice treated with the indicated treatments, with or without CD4^+^ or CD8^+^ T cells depletion (n = 6). αCD4, anti-mouse CD4 depletion antibody; αCD8, anti-mouse CD8 depletion antibody. (H-K) Anti-CD3ε/CD28-stimulated OVA-specific CD8⁺ T cells were co-cultured with BCR-ABL1⁺ B-ALL cells pretreated with the indicated treatments in the presence of OVA peptide. A bar graph illustrates the percentage of Ki-67⁺ CD8⁺ T cells (H). A histogram shows CD69 expression on CD8⁺ T cells, along with the percentage of CD69⁺ CD8⁺ T cells (I). A histogram displays CD25 expression on CD8⁺ T cells, as well as the percentage of CD25⁺ CD8⁺ T cells (J). A bar graph shows the percentage of IFN-γ⁺ CD8⁺ T cells (K). Data represent three independent experiments. (L) Reverse transcription quantitative polymerase chain reaction (RT-qPCR) was used to detect MHC-I expression in BCR-ABL1^+^ B-leukemia cells. MHC-I mRNA fold expression values have been normalized to the β-actin. Data represent three independent experiments. (M) Western blotting was used to detect MHC-I expression in BCR-ABL1^+^ B-leukemia cells. The same blot was reprobed with an anti-β-actin antibody to assess protein loading. Data represent 3 independent experiments. (N) Flow cytometry was used to assess the efficiency of *B2m* knockout in BCR-ABL1^+^ B-ALL cells. (O) Kaplan-Meier survival curves of WT and *B2m* KO BCR-ABL1^+^ B-ALL mice (n = 4). (P) Kaplan-Meier survival curves of *B2m* knockout BCR-ABL1^+^ B-ALL mice treated with vehicle, a combination of IM with 17-DMAG, or IM with CD19@NP/17-DMAG (n = 5). Unless otherwise stated, results are presented as the means ± SEM: *P < 0.05, **P < 0.01, ***P < 0.001 and ****P < 0.0001. Stars indicate a significant *p* value as calculated by the relevant statistical test.

Previous studies have shown that CD4⁺/CD8⁺ T cell exhaustion occurs rapidly after leukemia establishment in both murine B-ALL models and human patient samples 41, 42. To determine whether CD19@NP/17-DMAG and imatinib could reverse this exhausted T cell phenotype in BCR-ABL1⁺ B-ALL, we administered the indicated treatments beginning on day 8 post-leukemia cell injection (Figure 4A). One week later, the mice were euthanized for analysis. Treatment with CD19@NP/17-DMAG and imatinib significantly altered the immune cell composition in the spleen, notably increasing the numbers of total T cells, dendritic cells, and macrophages. Importantly, exhaustion in both CD4⁺ and CD8⁺ T cells was reduced, leading to higher activation marked by increased IFN-γ secretion. In addition, the combination therapy substantially lowered the proportion of Tregs compared to other treatments (Figure 6A-F, Figure S6A-C), and further improved the Treg/CD8⁺ T and Treg/CD4⁺ T ratios (Figure S6D-E). These findings suggest that CD19@NP/17-DMAG effectively stimulates T-cell responses against BCR-ABL1⁺ B-ALL. Moreover**,** to evaluate the roles of CD4⁺ and CD8⁺ T cells in treatment efficacy, each subset was depleted prior to therapy initiation in separate groups. Depletion of either subset markedly diminished the protective effect of CD19@NP/17-DMAG but did not affect the efficacy of 17-DMAG alone (Figure 6G, Figure S6F). These results indicate that CD19@NP/17-DMAG enhances therapeutic outcomes by activating both CD4⁺ and CD8⁺ T cells, highlighting its immunomodulatory potential.

### Enhanced MHC-I-mediated antigen presentation underlies improved CD8^+^T activation in BCR-ABL1⁺ B-ALL treated with CD19@NP/17-DMAG and Imatinib

Our RNA-seq analysis revealed that CD19@NP/17-DMAG treatment altered the expression of genes involved in antigen processing and presentation (Figure 5G). We hypothesized that this targeted therapy activates pathways in BCR-ABL1⁺ B-ALL cells that enhance antigen processing and presentation. To test this, OVA-specific CD8⁺ T cells from the spleen of OT-1 mice were co-cultured with BCR-ABL1⁺ B-ALL cells pretreated with either CD19@NP/17-DMAG plus imatinib or 17-DMAG plus imatinib, both in the presence of OVA peptide.

The OVA-specific CD8⁺ T cells co-cultured with the CD19@NP/17-DMAG plus imatinib-pretreated B-ALL cells exhibited greater proliferation and activation than those co-cultured with the 17-DMAG plus imatinib-pretreated B-ALL cells (Figure 6H-K, Figure S6G-H). Furthermore, we confirmed that CD19@NP/17-DMAG plus imatinib treatment increased MHC-I expression in BCR-ABL1⁺ B-ALL cells compared to treatment with 17-DMAG plus imatinib or the vehicle control (Figures 6L-M). Since MHC-I expression on tumor cells is crucial for CD8⁺ T cell activation 43, its loss can facilitate immune escape and resistance to immune checkpoint therapy 44-47. To determine whether MHC-I was necessary for the treatment's effectiveness, we knocked out the *B2m* gene in BCR-ABL1⁺ B-ALL cells using CRISPR/Cas9, confirming MHC-I loss by flow cytometry (Figure 6N). *In vivo* studies using both wild-type and *B2m*-knockout B-ALL cells showed that the loss of MHC-I did not affect disease onset or progression (Figure 6O). However, when *B2m*-knockout BCR-ABL1⁺ B-ALL mice were treated, the combination of imatinib and CD19@NP/17-DMAG only slightly extended survival by 4 days over the vehicle control and by 2 days over imatinib plus 17-DMAG (Figure 6P). Taken together, these findings suggest that CD19@NP/17-DMAG plus imatinib treatment increases MHC-I expression and antigen presentation in BCR-ABL1⁺ B-ALL cells, which is essential for robust CD8⁺ T cell-mediated responses.

### CD19@NP/17-DMAG combined with the broad-spectrum TKI ponatinib achieves long-term remission and establishes anti-leukemia immune memory in BCR-ABL1^+^ B-ALL

**Figure 7:**
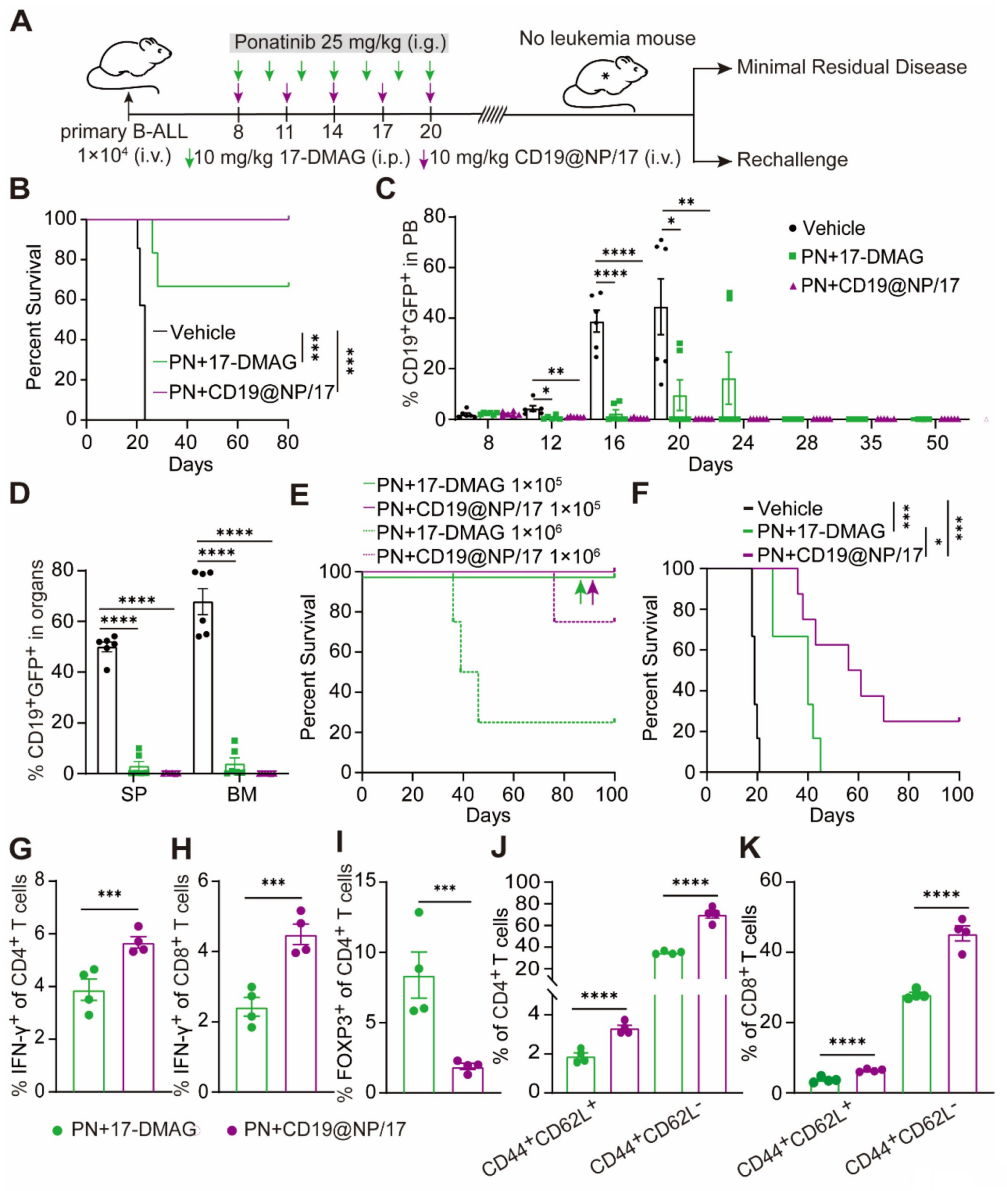
Eradication of minimal residual disease and elicitation of protective memory response in BCR-ABL1^+^ B-ALL mice treated by CD19@NP/17-DMAG with ponatinib. (A) Overview of the indicated treatment options for BCR-ABL1^+^ B-ALL mice. (B) Kaplan-Meier survival curves of BCR-ABL1^+^ B-ALL mice treated with vehicle, or a combination of ponatinib (PN) with 17-DMAG, or CD19@NP/17-DMAG (n = 6). (C) Monitoring of CD19^+^ GFP^+^ cell percentages in peripheral blood of BCR-ABL1^+^ B-ALL mice treated with the indicated treatments at specified time points (n = 6). (D) Percentage of CD19^+^ GFP^+^ cells in spleen (SP) and bone marrow (BM) following the indicated treatments on day 16 (n = 6). (E) Kaplan-Meier survival curves of secondary mice transplants with the indicated dilutions of BM cells treated with combinations of PN either 17-DMAG or CD19@NP/17-DMAG (n = 5). Colored arrows indicate overlapping corresponding curves. (F) Kaplan-Meier survival curves of re-challenge experiments for the indicated groups (n = 6-8). Naïve C57BL/6 mice injected with 1 × 10⁵ leukemia cells served as positive controls. Mice previously cured (surviving over 100 days) by treatment with PN and CD19@NP/17-DMAG were re-challenged via transplantation with a tenfold higher dose of leukemia cells compared to the initial injection. Disease progression was monitored based on survival, without any further treatment. (G-I) Bar graphs show the percentages of IFN-γ^+^ CD4^+^ T cells (G), IFN-γ^+^ CD8^+^ T cells (H), and Treg cells (I) in the spleens of BCR-ABL1^+^ B-ALL mice (n = 4) on day 9 following leukemia cell re-challenge with the indicated treatments. (J-K) Bar graphs illustrate the percentages of CD4^+^ (J) and CD8^+^ (K) central and effector memory T cells in the spleens of BCR-ABL1^+^ B-ALL mice (n = 4) as indicated. Unless otherwise stated, results are presented as the means ± SEM: *P < 0.05, **P < 0.01, ***P < 0.001 and ****P < 0.0001. Stars indicate a significant *p* value as calculated by the relevant statistical test.

Despite initial remission with imatinib and CD19@NP/17-DMAG, all mice eventually relapsed (Figure 4B). Previous studies suggest that mutations in the BCR-ABL1 tyrosine kinase domain can cause relapse in Ph^+^ ALL mouse models treated with BCR-ABL1-targeted TKIs like imatinib 2, 48. However, Sanger sequencing showed no mutations in the BCR-ABL1 tyrosine kinase domain in mice treated with imatinib plus 17-DMAG or CD19@NP/17-DMAG (Table S1). This suggests that disease relapse was due to other mechanisms, despite a reduction in BCR-ABL1 expression and kinase activity (Figure 4H, Figure 5H). Given the ability of CD19@NP/17-DMAG to stimulate T-cell responses and induce leukemia cell apoptosis, we hypothesized that combining it with the broad-spectrum TKI ponatinib could maximize efficacy 49-52. Using a similar approach as before (Figure 7A), we treated mice with CD19@NP/17-DMAG combined with ponatinib. Remarkably, 100% of mice achieved relapse-free survival up to 80 days after treatment, with leukemic cells becoming undetectable in the blood, bone marrow, and spleen (Figure 7B-D). T-cell numbers increased, and IFN-γ-secreting CD4^+^ and CD8^+^ T cells also rose (Figure S7A-C). Meanwhile, the number of exhausted CD4^+^ and CD8^+^ T cells decreased dramatically (Figure S7D-E), and regulatory T cells (Tregs) declined sharply (Figure S7F).

To assess the effectiveness of this combination in clearing minimal residual disease, mice treated with either ponatinib plus 17-DMAG or ponatinib plus CD19@NP/17-DMAG were monitored for relapse-free survival over four weeks. Bone marrow cells from these mice were then transplanted into secondary recipients. Ponatinib combined with either 17-DMAG or CD19@NP/17-DMAG extended survival beyond 100 days in recipients receiving 1 × 10^5^ bone marrow cells. Notably, 75% of recipients receiving 1 × 10^6^ bone marrow cells from ponatinib plus CD19@NP/17-DMAG-treated mice survived beyond 70 days, compared to only 25% from the ponatinib plus 17-DMAG group (Figure 7E).

Building on these results, we investigated whether CD19@NP/17-DMAG with ponatinib could induce long-term immune memory. In a re-challenge experiment, mice cured of leukemia were re-injected with a ten-fold higher dose of leukemic cells, while naïve mice served as controls. Remarkably, 25% of mice treated with ponatinib plus CD19@NP/17-DMAG mounted a protective memory response, preventing leukemia re-establishment over 100 days, however all mice treated with ponatinib plus 17-DMAG eventually succumbed to leukemia (Figure 7F). In post-re-challenge experiments, treated mice showed increased IFN-γ production in CD4^+^ and CD8^+^ T cells and reduced Tregs (Figure 7G-I). Furthermore, central and effector memory T-cell levels increased significantly, aligning with higher cure rates (Figure 7J-K). These results suggest that the combination of ponatinib and CD19@NP/17-DMAG not only triggers an anti-leukemia T-cell response but also promotes T-cell memory development, enhancing long-term immune surveillance against leukemia.

### CD19@NP/17-DMAG demonstrates efficacy against A20 lymphoma by enhancing the T cell immune response

**Figure 8:**
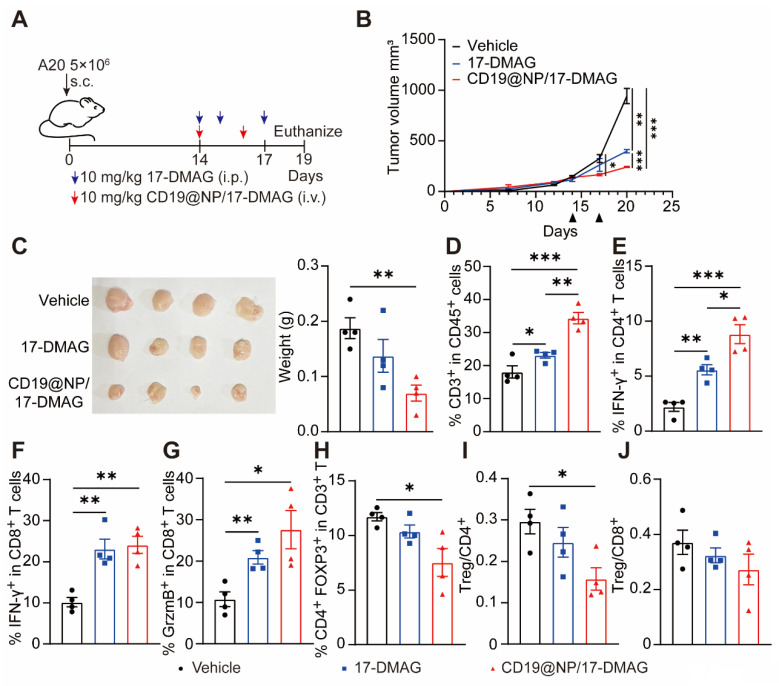
CD19@NP/17-DMAG treatment demonstrates efficacy against subcutaneous A20 lymphoma. (A) Overview of treatment options for subcutaneous A20 lymphoma. (B) The tumor growth rate after the indicated treatments (n = 4). (C) Tumors were excised from mice in the indicated treatment groups (n = 4). The tumor weights are plotted, with each dot representing a tumor from one mouse. (D-J) Analysis of different T cell populations from subcutaneous tumors (n = 4) after one week of treatment. Bar graphs illustrate the percentages of total CD3^+^ T cells (D), IFN-γ^+^ CD4^+^ T cells (E), IFN-γ^+^ CD8^+^ T cells (F), GrzmB^+^ (Granzyme B) CD8^+^ T (G) cells and Treg cells (H), along with (I) the Treg/CD4^+^ and (J) Treg/CD8^+^ ratios. Error bars indicate mean ± SEM. Statistical significance is indicated as follows: *P < 0.05, **P < 0.01, ***P < 0.001 and ****P < 0.0001.

To further test the effectiveness of the CD19@NP/17-DMAG drug delivery system in other B-cell malignancies, we used a mouse A20 lymphoma model 47. We transplanted 5 × 10^6^ A20 lymphoma cells into syngeneic BALB/c mice via subcutaneous injection. When the tumors reached 100 to 200 mm^3^, mice were divided into three groups and treated with either 17-DMAG or CD19@NP/17-DMAG for one week (Figure 8A). In this model, CD19@NP/17-DMAG treatment significantly slowed tumor growth compared to the other groups (Figure 8B-C). Analysis of tumor cell suspensions 72 h after the last treatment showed that CD19@NP/17-DMAG significantly increased the number of CD3^+^ T cells infiltrating the tumor (Figure 8D).

We also observed a higher number of IFN-γ-producing CD4^+^ and CD8^+^ T cells in the CD19@NP/17-DMAG-treated mice compared to the other groups (Figure 8E-F). Furthermore, CD19@NP/17-DMAG treatment increased the proportion of granzyme B-secreting CD8^+^ T cells (Figure 8G). It also reduced the number of infiltrating regulatory T cells (Tregs) and lowered the Treg/CD8^+^ T-cell and Treg/CD4^+^ T-cell ratios (Figure 8H-J). These results indicate that the effectiveness of CD19@NP/17-DMAG therapy in treating B-cell lymphomas relies on a stronger T-cell immune response, making it a potentially more effective approach than traditional treatments.

## Discussion

One of the major challenges in treating B-cell malignancies is overcoming drug resistance and managing the severe side effects associated with conventional therapies such as chemotherapy, monoclonal antibodies, and CAR-T cell therapies, which often lead to myelosuppression, immune complications, and disease relapse. To address these issues, we developed a novel therapeutic strategy using CD19-targeted nanoparticles loaded with the HSP90 inhibitor 17-DMAG for the treatment of B-cell malignancies. In mouse models of BCR-ABL1^+^ B-ALL and B-cell lymphoma, CD19@NP/17-DMAG, both alone and in combination with TKIs like imatinib and ponatinib, demonstrated significant efficacy by reducing tumor burden, prolonging survival, and activating a strong anti-tumor T-cell response. Notably, the upregulation of tumor-derived MHC-I expression enhanced antigen presentation facilitating more effective recognition of cancer cells by cytotoxic T lymphocytes, thereby improving treatment efficacy. These findings suggest that CD19@NP/17-DMAG is a promising therapy for B-cell malignancies, offering both immediate tumor control and durable immune protection while potentially minimizing the side effects associated with conventional treatments.

We developed a biocompatible, safe, and effective drug delivery system by designing CD19-targeted PLGA-NHS nanoparticles loaded with 17-DMAG. PLGA nanoparticles are known for their biodegradability, biocompatibility, and sustained drug release properties, but their limited surface functional groups restrict targeting ligand attachment. To overcome this limitation, we incorporated PLGA with PLA-PEG-NHS, enabling covalent attachment of targeting ligands via NHS modification, resulting in PLGA-NHS nanoparticles. Unlike other systems, such as PLA-PEG-FA, which target cells overexpressing folate receptors, PLGA-NHS nanoparticles are more versatile and have broader targeting potential 33, 38. We demonstrated that recombinant CD19 scFv successfully conjugated to PLGA-NHS nanoparticles, resulting in the new formulation, CD19@NP. Characterization of CD19@NP showed an increase in particle diameter compared to PLGA-NHS, confirming successful scFv coating. Additionally, the shift in zeta potential to a near-neutral charge indicates effective surface functionalization (Figure 2). This near-neutral charge improves interactions with biological membranes and cells, enhancing targeting, though it may reduce electrostatic repulsion between particles, potentially decreasing colloidal stability and increasing aggregation risk 36, 37, 53, 54. Given the physical properties of CD19@NP, we analyzed their phagocytosis and *in vivo* biodistribution. Compared to PLGA-NHS, myeloid cells demonstrated increased phagocytosis of CD19@NP, suggesting a potential trade-off between targeted delivery and immune clearance 55. Nonetheless, *in vivo*, CD19@NP accumulated in lymphoid organs (e.g., bone marrow, spleen, lymph nodes) and blood-rich organs (e.g., liver), indicating effective targeting for immune and hematological diseases such as leukemia or lymphoma. Most importantly, CD19@NP is biosafe extended the metabolic half-life *in vivo* (Figure 3). These findings underscore the need to optimize the balance between targeting efficacy and immune clearance to design an appropriate therapeutic window for CD19@NP.

The targeted delivery system significantly enhanced the therapeutic effects of 17-DMAG. The HSP90 inhibitor 17-DMAG disrupts the chaperone activity of HSP90, which is crucial for the function of multiple oncogenic proteins, leading to the degradation of various client proteins and inducing tumor cell apoptosis. Compared to our previous data using the combination of free 17-DMAG and imatinib, the CD19-targeted nanoparticle delivery system with imatinib significantly reduced disease burden and improved survival in a murine model of BCR-ABL1^+^ B-ALL (Figure 4). Moreover, the expression levels of key HSP90 client proteins, BCR-ABL1 and AID, were markedly lower in the group treated with CD19@NP/17-DMAG and imatinib. The reduction of BCR-ABL1 disrupts the primary oncogenic driver in Ph^+^ B-ALL, while decreased AID levels may prevent genomic instability and resistance development 23, 56. These findings suggest that the targeted delivery system amplifies the inhibitory effects on critical survival pathways in leukemia cells.

To elucidate the molecular mechanisms underlying these enhanced therapeutic effects, we performed RNA-seq analysis on GFP^+^ CD19^+^ leukemic cells post-treatment. The CD19@NP/17-DMAG combined with imatinib impacted a broader range of signaling pathways compared to the combination of free 17-DMAG and imatinib, resulting in significant alterations in pathways related to cell differentiation, proliferation, and apoptosis. Interestingly, the targeted delivery also affected various metabolic pathways, including glycolysis/gluconeogenesis and amino acid metabolism (Figure 5). Cancer cells often rely on altered metabolism for rapid growth and survival 57, 58. By disrupting these metabolic processes, CD19@NP/17-DMAG may further sensitize leukemic cells to apoptosis and enhance the overall therapeutic effect.

Importantly, CD19-targeted 17-DMAG nanoparticles, either combined with the BCR-ABL1-targeted TKI imatinib or the broad-spectrum TKI ponatinib 51, 52, not only reduced tumor burden but also significantly enhanced T-cell immunity in BCR-ABL1^+^ B-ALL. Both treatments effectively reversed the exhausted phenotype and reactivated CD4^+^ and CD8^+^ T cells. Furthermore, depletion of CD4^+^ or CD8^+^ T cells directly supported that both T-cell subsets are crucial for the therapeutic efficacy of CD19@NP/17-DMAG. This aligns with previous research highlighting the pivotal roles of CD4^+^ helper T cells and CD8^+^ cytotoxic T cells in controlling leukemia progression 59, 60. Our data indicated that CD19@NP/17-DMAG significantly boosted the T-cell immune response compared to CD19@NP alone (data not shown). These findings align with emerging research highlighting the importance of immunogenic cell death (ICD) in cancer therapy. ICD can release tumor antigens and danger-associated molecular patterns (DAMPs), promoting dendritic cell maturation and T-cell activation 61-64. Specifically, our results suggest that HSP90 inhibitors like 17-DMAG may induce ICD, and when delivered directly to tumor cells via CD19-targeted nanoparticles, this effect can be further amplified. Furthermore, we observed increased antigen presentation and upregulated MHC-I expression on tumor cells following treatment (Figure 6), enhancing their susceptibility to T-cell-mediated killing.

Despite achieving initial remission with imatinib and CD19@NP/17-DMAG, all mice eventually relapsed. However, the combination of CD19@NP/17-DMAG and ponatinib achieved 100% relapse-free survival up to 80 days post-treatment, eradicating minimal residual disease, activating a robust antitumor T-cell immune response, and fostering long-term immune memory (Figure 7). This suggests a more effective eradication of leukemia cells. Compared to imatinib, the enhanced efficacy of CD19@NP/17-DMAG with the broad-spectrum TKI may stem from increased exposure of tumor-specific neoantigens, eliciting a highly specific anti-tumor T-cell response 65-67. Furthermore, these findings offer valuable insights for potentially developing a tumor vaccine for BCR-ABL1^+^ B-ALL, where the immunogenic effects of the CD19@NP/17-DMAG system could be harnessed to induce durable T-cell memory as a safeguard against disease recurrence.

Extending the application of the CD19@NP/17-DMAG delivery system, we demonstrated its efficacy in another B-cell malignancy model, the A20 lymphoma. Treatment with CD19@NP/17-DMAG significantly slowed tumor growth and enhanced also reshapes the immune landscape to favor anti-tumor activity. This underscores the potential of CD19@NP/17-DMAG as a promising therapeutic strategy for B-cell lymphomas and other CD19-expressing malignancies.

In conclusion, the CD19@NP/17-DMAG system represents a promising therapeutic strategy for B-cell malignancies by offering targeted delivery, reduced toxicity, and the potential to elicit a robust anti-tumor immune response. These findings underscore the potential of CD19@NP/17-DMAG for treating B-cell lymphomas and other CD19-expressing malignancies. Future studies should further elucidate the signaling pathways involved in CD8⁺ T cell activation and explore combination regimens with other immunotherapies, such as immune checkpoint inhibitors, to maximize therapeutic efficacy for B-cell malignancies.

## Supplementary Material

Supplementary methods and figures.

## Figures and Tables

**Table 1:** Characterization of PLGA and PLGA-NHS nanoparticles

Index	PLGA	PLGA-NHS
Z-Average	287.2 ± 0.931	220.7 ± 0.964
PDI	0.104	0.04
Zeta	-15.9 ± 5.54	-23.2 ± 6.41

**Table 2:** Drug loading and encapsulation efficiency of PLGA-NHS at various dose of 17-DMAG

17-DMAG	Encapsulation efficiency (%)	Loading efficiency (%)
2 mg	75 ± 0.07	2.8 ± 0.56
5 mg	60 ± 0.05	5.5 ± 0.73
10 mg	65 ± 0.07	10.8 ± 0.98
15 mg	52 ± 0.08	7.7 ± 0.52

## Abbreviations

HSP90: heat shock protein 90
BCR-ABL1: breakpoint cluster region-abelson1
scFv: single-chain variable fragment
TKIs: tyrosine kinase inhibitors
MHC-I: major histocompatibility complex class I
RNA-seq: RNA sequencing
RT-qPCR: reverse transcription quantitative polymerase chain reaction
