# Supplementary material for: CD19-targeted HSP90 inhibitor nanoparticle combined with TKIs reduces tumor burden and enhances T-cell immunity in murine B-cell malignancies
Source: Theranostics. 2025 Feb 25;15(8):3589–609. doi: 10.7150/thno.106758 (PMC11905126; doi:10.7150/thno.106758)
Supplement: Supplementary file 1 — Supplementary methods and figures. [file thnov15p3589s1.pdf]

Supplemental material

Supplemental Experimental Methods

H&E Staining

After euthanasia, heart, liver, spleen, lung, and kidney tissues were collected from mice and fixed in 10% buffered formalin for 72 h at room temperature. The fixed tissues were then embedded in paraffin blocks. Sections of 5 μm thickness were cut from these paraffin-embedded tissues and stained with hematoxylin and eosin (H&E) (Solarbio, China), following the manufacturer's instructions. The stained tissue sections were imaged using a Zeiss Axio Scope A1 microscope (Carl Zeiss Microscopy GmbH).

Flow cytometry

Immune cells were analyzed by flow cytometry using CytoFLEX (Beckman Coulter, Inc.). Cells were stained with one or more of the following antibodies from BioLegend. APC anti-mouse/human CD44 (103012), PE anti-mouse CD62L (104408), PerCP/Cyanine5.5 anti-mouse CD4 (100434), APC anti-mouse CD366 (Tim-3) (119705), APC anti-mouse CD366 (Tim-3) (134008), PE anti-mouse c-kit (105805), FITC anti-mouse CD45 (103108), APC anti-mouse sca-1 (108111), FITC anti-mouse NK1.1 (108708), PE anti-mouse CD11c (117308), PE/Cyanine7 anti-mouse CD11b (101216), PerCP/Cyanine5.5 anti-mouse F4/80 (123128), PE anti-mouse Ly-6G (127608), Pacific Blue™ anti-mouse Ly-6C (128031), APC anti-mouse Granzyme B (372204), Pacific Blue™ anti-mouse CD3 (100214), PE/Cyanine7 anti-mouse CD8b (126616), APC anti-mouse CD45R/B220 (103212), APC anti-mouse CD19 (159806), PE anti-mouse CD279 (PD-1) (135206), PE anti-mouse IFN-γ (163504), PE anti-mouse FOXP3 (118904), PE Streptavidin (405203) ,PE anti-mouse CD69 (104507), Brilliant Violet 421™ anti-mouse CD25 (113705), APC anti-mouse Ki-67 (652406) and

PE anti-mouse H-2K^b (116507). Cells were stained with PerCP-Cy™5.5 Mouse Lineage Antibody Cocktail (51-9006964) from BD Pharmingen™. Succinimidyl D-biotin (1555261) was purchased from Thermo Fisher. The anti-CD3ε (100340) and anti-CD28 (102116) antibodies used to stimulate T cell proliferation were purchased from BioLegend. Data was analyzed with Kaluza Analysis Software 2.1 (Beckman Coulter, Inc.).

Western blotting

Protein extraction was performed using NP-40 lysis buffer (Solarbio, China), supplemented with protease and phosphatase inhibitors. Proteins (60 μg per lane) were separated by 10% SDS-PAGE and subsequently electro-transferred onto 0.45 μm polyvinylidene difluoride (PVDF) membranes (Millipore, Immobilon-P, IPVH00010). The membranes were blocked with 5% skimmed milk for 2 h at room temperature and then incubated overnight at 4°C with primary antibodies including rabbit anti-c-Abl (2862, Cell Signaling Technology), rat anti-AID (14-5959-82, Invitrogen), rabbit anti-phospho-CrkL (ab52908, Abcam), rabbit anti-CrkL (ab32126, Abcam), mouse anti-Hsp90 (ab13492, Abcam), rabbit anti-phospho-Akt (4060, CST), rabbit anti-Akt (4691, CST), rabbit anti-phospho-Btk (87141, CST), rabbit anti-Btk (3533, CST), rabbit anti-phospho-NF-κB p65 (3033, CST), rabbit anti-NF-κB p65 (8242, CST), rabbit anti-phospho-Erk1/2 (8544, CST), rabbit anti-Erk1/2 (9102, CST), rabbit anti-phospho-Stat5 (4322, CST), rabbit anti-Stat5 (94205, CST), anti-β-actin (8457, CST), rabbit anti-Mcl-1 (5453, CST), rabbit anti-Bcl-xL (2764, CST), rabbit anti-Bcl-2 (3498, CST), rabbit anti-Bad (ab32445, Abcam), mouse anti-Caspase-3 (ab13585, Abcam), mouse anti-Cytochrome C (ab13575, Abcam), rabbit anti-Ku-80 (YT6205, Immunoway) and rabbit anti-MHC class I (35923, CST). Subsequently, the membranes were incubated with HRP-conjugated secondary antibodies: goat anti-rabbit IgG (31466, Thermo Fisher Scientific), goat anti-rat IgG (31470, Thermo Fisher Scientific), or goat anti-mouse IgG

(31431, Thermo Fisher Scientific) for 45 min at room temperature. The blots were visualized using Western Lightning Pro ECL (Fusion FX5, Vilber).

BrdU proliferation and apoptosis assays

1 mg of BrdU reagent was intraperitoneally injected into mice. One hour later, the mice were euthanized, and spleen tissues were collected. The tissues were then stained using a BrdU kit (552598, BD Biosciences) and an apoptosis detection kit (559763, BD Biosciences) according to the manufacturers' instructions. Flow cytometric data were analyzed using Kaluza Analysis Software 2.1 (Beckman Coulter, Inc.).

Mutations in ABL1 kinase domain

Genomic DNA was extracted from 5×10^6 cells using the DNA Extraction Kit (D824A, TaKaRa), following the manufacturer's instructions. The ABL1 kinase portion of the BCR-ABL1 gene was amplified through a two-round PCR process. In the first round, fragments of BCR-ABL1 were amplified using primers specific to BCR (exon 13) and ABL1 (exon 9), to minimize co-amplification of the normal ABL1 gene. The primers used were BCR exon 13 F: 5'-TTCAGAAGCTTCTCCCTGACAT-3' and ABL1 exon 9 R: 5'-CTTCGTCTGAGATACTGGATTCCT-3'. In the second round, the amplification focused on the ABL1 kinase domain (exons 3-8) using the primers ABL1 exon 3 F: 5'-GTGCGTGAGAGTGAGAGCAG-3' and ABL1 exon 8 R: 5'-GGTAGCAATTTCCCAAAGCA-3'. High fidelity DNA polymerase KOD-Plus-Neo (KOD-401, Toyobo Life Science) was used for the amplification. The PCR products were then purified using the Gel Extraction Kit (D2500-01, Omega Bio-Tek) and cloned into the pMD®18-T vector (6011, TaKaRa). The clones were subsequently sequenced by Sunny Biotech Co. Ltd.

Quantitative RT-PCR

Total RNA was extracted from splenic leukemia cells using TRIzol (Invitrogen; Thermo Fisher Scientific, Inc.) according to the manufacturer's instructions. The cDNA was generated from the total RNA using the PrimeScript™ RT reagent Kit (TaKaRa Bio, Inc.) following the manufacturer's protocol. Quantitative PCR experiments were performed in triplicate with SYBR-Green dye (TaKaRa Bio, Inc.) on an Mx3000P qPCR system (Agilent Technologies, Inc.). All data were analyzed using the $2^{(-\Delta\Delta Cq)}$ method (relative quantification). Gene expression was normalized to GAPDH. The primer sequences used for quantitative PCR were: MHC class I Forward: 5'-CGTTGCTGTTCTGGTTGTCCTTG-3', MHC class I Reverse: 5'-TCCACCTGTGTTTC TCCTTCTCATC-3'

Supplemental tables

Table S1. Identity of BCR-ABL1 kinase domain mutations

Therapy	Number of mice	Mutation
Vehicle	4	None
IM+17-DMAG	10	None
IM+CD19@NP/17-DMAG	10	None

***Note**:* Leukemia cells were isolated from the spleens of mice in each treatment group by flow cytometry. Total genomic DNA was extracted from these cells, and PCR was performed using BCR-ABL1 primers. The PCR products were then subjected to Sanger sequencing to detect any mutations in the BCR-ABL1 gene following treatments.

Supplemental figures and figure legends

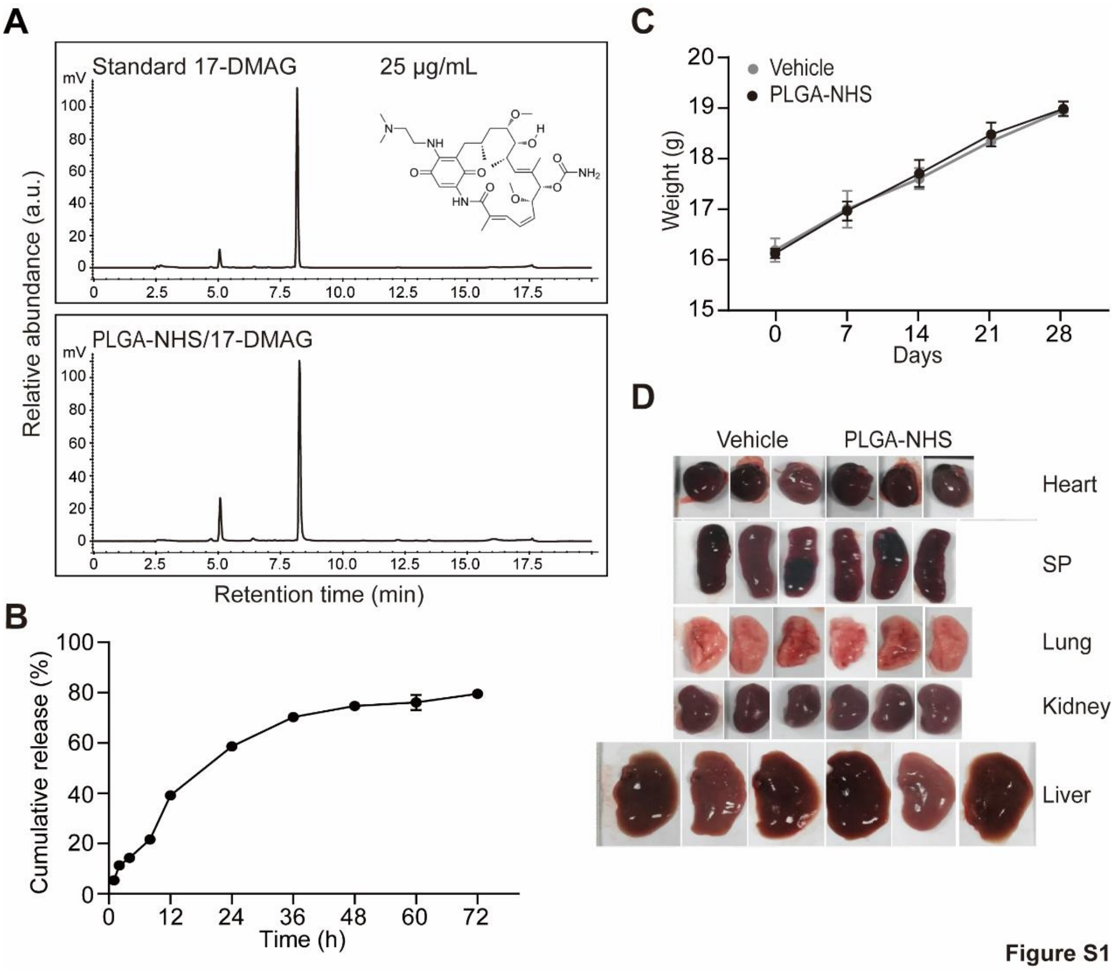

Figure S1. Characterization of 17-DMAG-loaded PLGA-NHS and biocompatibility of polymeric PLGA-NHS nanoparticles. (A) UV absorption peak of 17-DMAG at 25 µg/mL (top) and UV absorption peak of PLGA-NHS nanoparticles loaded with 10 mg of 17-DMAG (bottom), measured at 338 nm. A standard curve was generated using HPLC for 17-DMAG at varying concentrations. (B) Cumulative release profiles of 10 mg 17-DMAG from PLGA-NHS nanoparticles, evaluated at the indicated time points using HPLC and an in vitro dialysis technique. (C) Weight monitoring during the in vivo biocompatibility assay of PLGA-NHS nanoparticles in C57BL/6 mice (n = 3). (D) Representative images of the heart, liver, spleen, lung, and kidney from C57BL/6 mice treated with

either PLGA-NHS nanoparticles or vehicle control. Organs were harvested and analyzed from mice in both groups (n = 3).

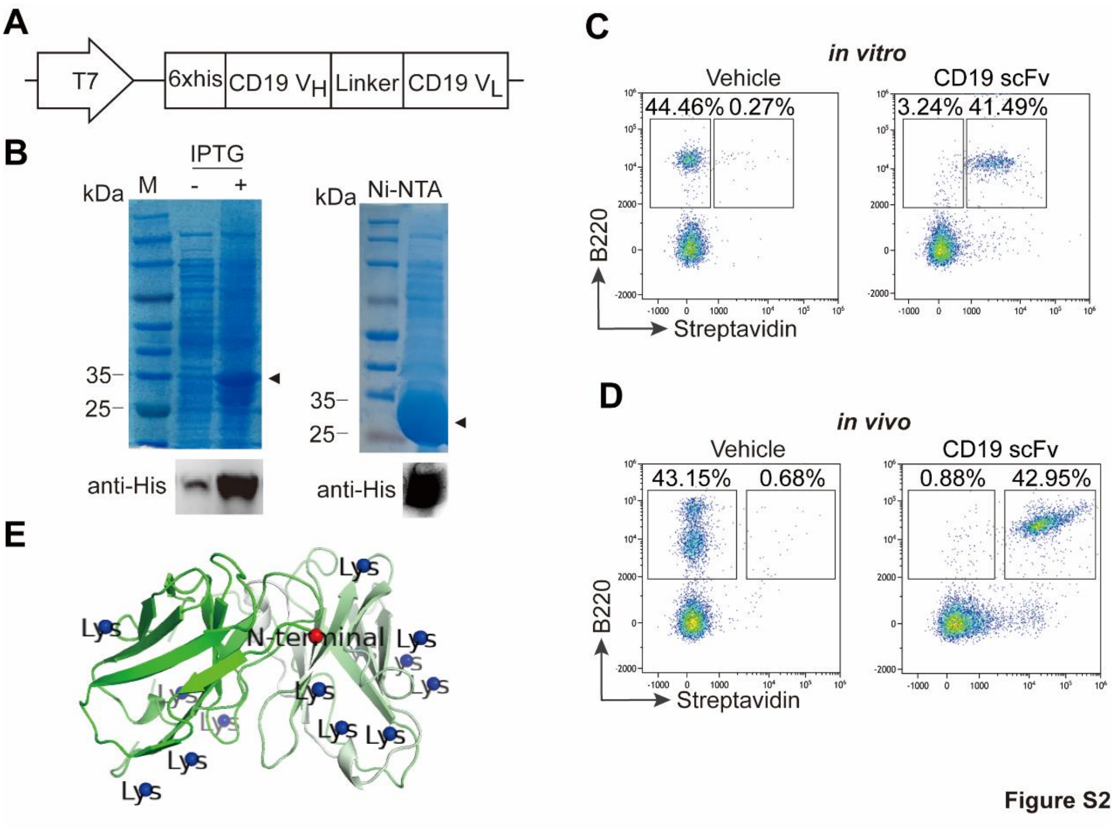

Figure S2. Preparation and validation of the targeting function of CD19 scFv. (A) Schema diagram of the recombinant murine CD19 scFv prokaryotic construct. V_H, anti-mouse CD19 single-chain variable fragment heavy chain; V_L, anti-mouse CD19 single-chain variable fragment light chain. (B) SDS-PAGE analysis of CD19 scFv expression in bacterial cell lysates. Bacteria were induced with 1 mM IPTG for 16 h at 25°C. Lane M: protein molecular weight marker; Lane '-': cell lysate supernatant from non-induced bacteria; Lane '+': lysate from IPTG-induced bacteria (left panel). CD19 scFv protein was purified using Ni-NTA affinity chromatography (right panel), and expression was confirmed by Western blotting with an anti-His antibody. (C) The targeting ability of CD19 scFv was examined in vitro by incubating splenocytes with biotin-labeled CD19

scFv, using free CD19 scFv as a negative control. After incubation, the cells were stained with anti-mouse B220 antibody and streptavidin, and the targeting ability was analyzed by flow cytometry. (D) The in vivo targeting ability of CD19 scFv was assessed by intravenously injecting mice with biotin-labeled CD19 scFv, with free CD19 scFv serving as a negative control. Peripheral blood samples were collected 10 min post-injection, and the cells were stained with anti-mouse B220 antibody and streptavidin, followed by analysis using flow cytometry. (E) The 3D structure of CD19 scFv was predicted using Phyre^2 software, with primary amine groups highlighted for detailed visualization. N-terminal amines are annotated with red spheres, while lysine side-chain amines are annotated with blue spheres.

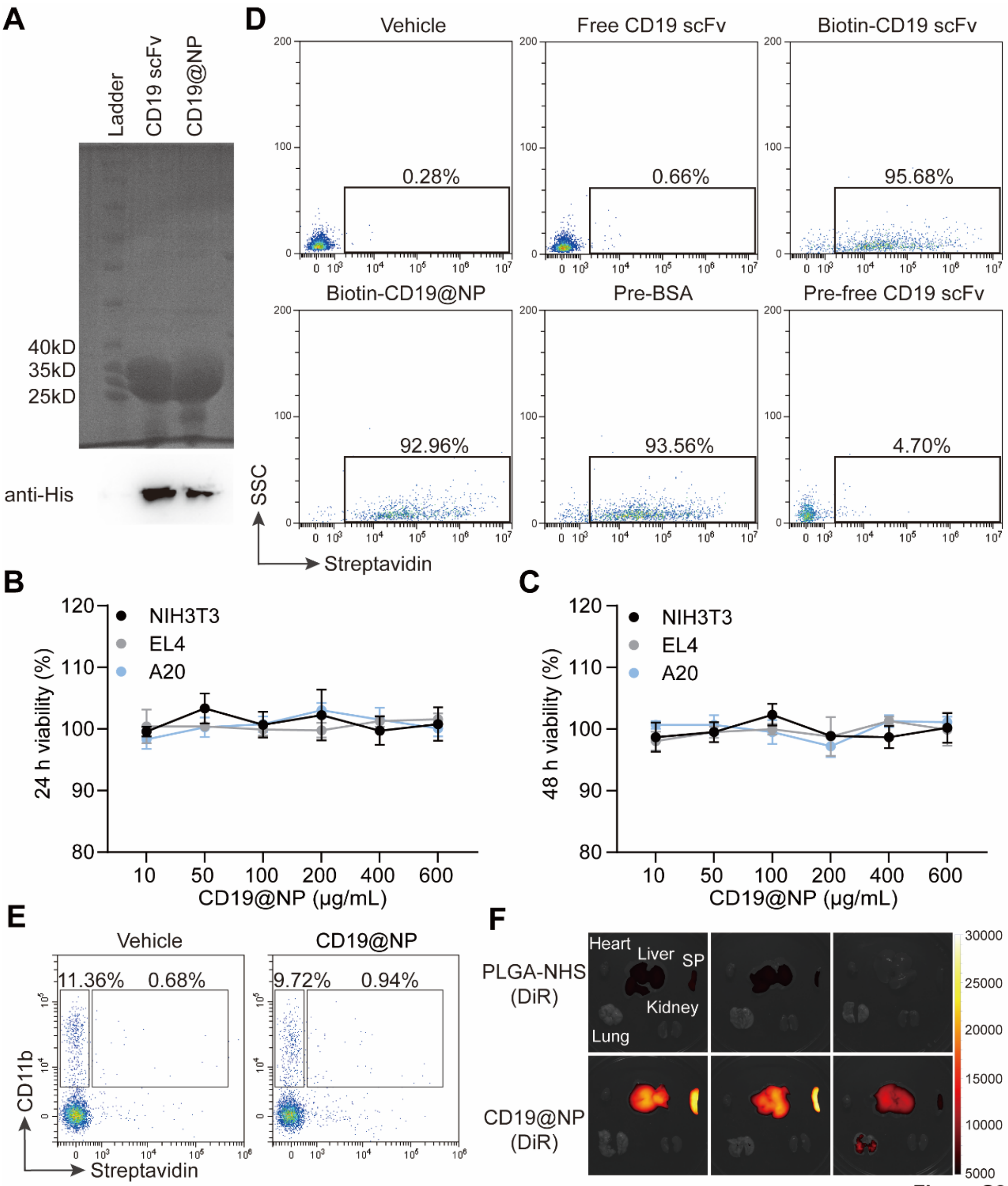

Figure S3

Figure S3. Characterization and validation of the specific targeting function of CD19@NP. (A) Coomassie Brilliant Blue staining (up panel) and Western blotting analysis of CD19@NP (down panel). (B-C) In vitro cytotoxicity assessment of CD19@NP nanoparticles on NIH/3T3, EL4 and A20 cells for 24 h (B) and 48 h (C) using the CCK-8 assay. Data represent the average of 3 independent experiments with error bars indicating the SEM. (D)The Competitive experiments of CD19@NP in pre-B

A70.2 cells. A70.2 cells were preincubated with either BSA or free CD19 scFv. This was followed by incubation with biotin-labeled CD19@NP. Meanwhile, cells incubated with PBS served as a blank control. Free CD19 scFv was used as a negative control. Biotin-labeled CD19 scFv and biotin-labeled CD19@NP were used as positive controls. All samples were subsequently stained with streptavidin and analyzed by flow cytometry. (E) The targeting ability of CD19@NP towards primary splenic myeloid cells from C57BL/6 mice in vitro. The targeting ability of CD19@NP was evaluated using primary splenic myeloid cells from C57BL/6 mice. The cells were incubated with biotin-labeled CD19@NP, while free CD19@NP was used as a negative control. After incubation, the cells were stained with anti-mouse CD11b and streptavidin, followed by analysis using flow cytometry. (F) Biodistribution of DiR-loaded CD19@NP in the heart, liver, spleen, lung, and kidney from healthy C57BL/6 mice (n = 3).

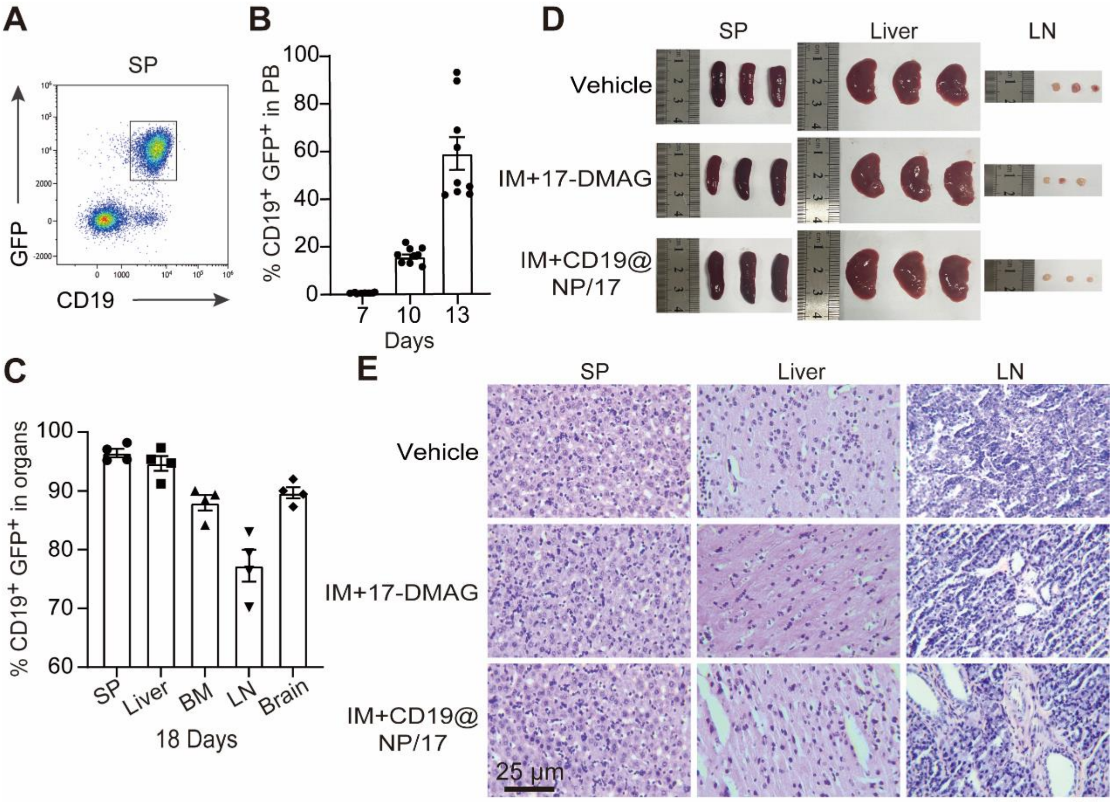

Figure S4

Figure S4. Reduction of leukemic burden in BCR-ABL1$^+$ B-ALL mice treated with CD19@NP/17-DMAG and imatinib. (A) A representative flow cytometry plot of spleen CD19$^+$ GFP$^+$ leukemic cells at day 18 post-injection. (B) Percentage of GFP$^+$ cells in the spleen (SP), liver, bone marrow (BM), lymph node (LN) and brain at day 18 post-injection of leukemia mice (n = 9). (C) Kinetic growth of leukemia cells was measured at the indicated timepoints (n = 4). Data are derived from 2 independent experiments. (D) Images of SP, liver and LN of mice with the indicated treatments (n = 3). (E) H&E staining of SP, liver and LN of mice with the indicated treatments (n = 3). The scale bar represents 25 μm.

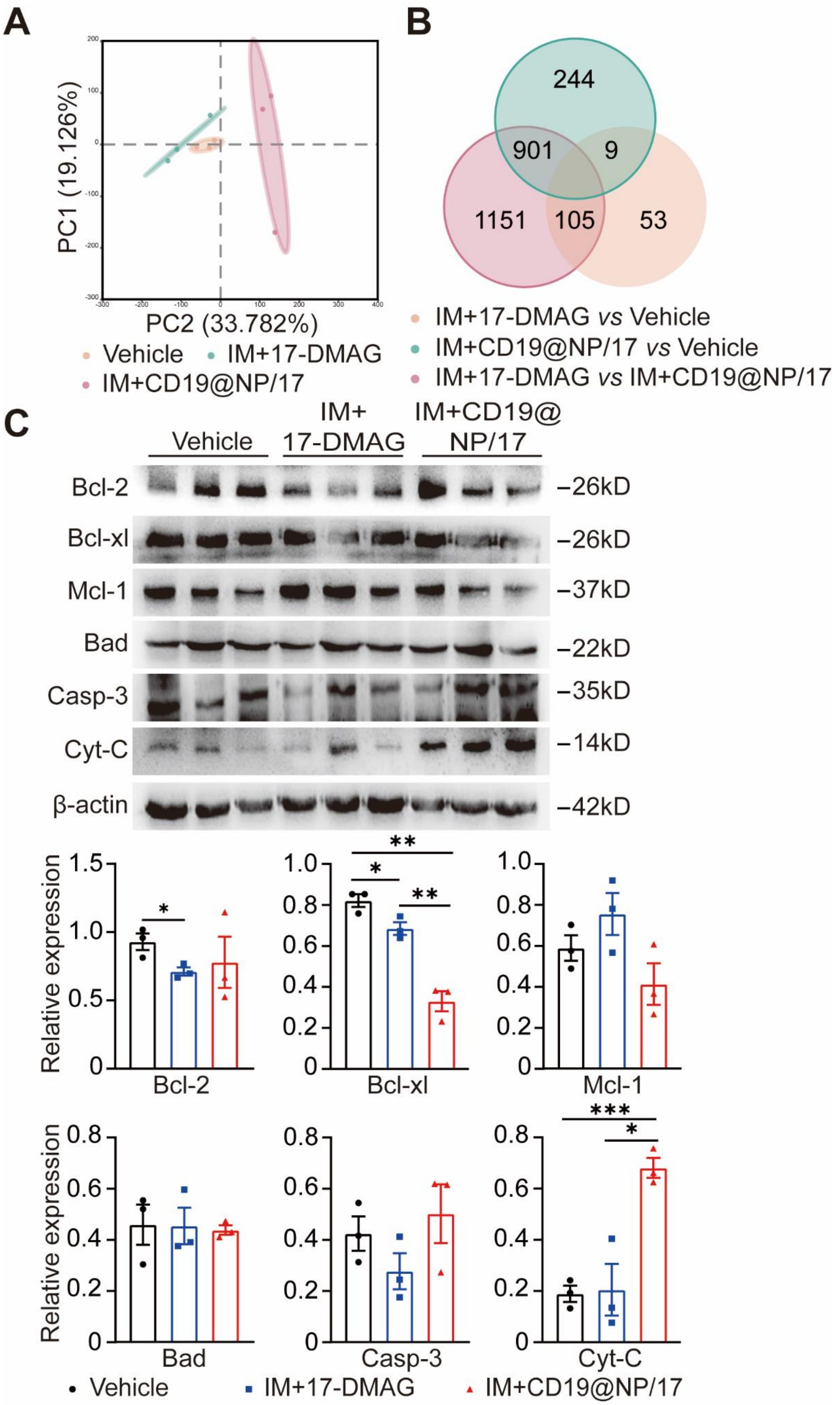
A
B
C
PC1 (19.126%)
PC2 (33.782%)
Vehicle
IM+17-DMAG
IM+CD19@NP/17
244
901
9
1151
105
53
IM+17-DMAG vs Vehicle
IM+CD19@NP/17 vs Vehicle
IM+17-DMAG vs IM+CD19@NP/17
Vehicle
IM+ 17-DMAG
IM+CD19@ NP/17
Bcl-2 –26kD
Bcl-xl –26kD
Mcl-1 –37kD
Bad –22kD
Casp-3 –35kD
Cyt-C –14kD
β-actin –42kD
Relative expression
Bcl-2
Bcl-xl
Mcl-1
Bad
Casp-3
Cyt-C
Vehicle
IM+17-DMAG
IM+CD19@NP/17

Figure S5

Figure S5. Validation of RNA-seq results via Western blotting. (A) Principal component analysis (PCA) plot illustrating the separation between the three groups based on their gene expression profiles. PC1 and PC2 account for 19.126% and 33.782% of the total variance, respectively. (B) Venn diagram showing the overlap of DEGs among the three pairwise comparisons. (C) Western blotting analysis of the decreased levels of anti-apoptotic proteins (Bcl-2, Bcl-xL, Mcl-1) and increased levels of pro-apoptotic proteins (Bad, Caspase-3, Cytochrome c), in BCR-ABL1$^+$ B-ALL leukemic cells from spleen of moribund deceased mice following the treatments (n = 3). The same blotting was reprobed with an anti-β-actin antibody to assess protein loading. Data represent three independent experiments. Error bars indicate mean ± SEM. Statistical significance is indicated as follows: *$P < 0.05$, **$P < 0.01$, ***$P < 0.001$ and ****$P < 0.0001$.

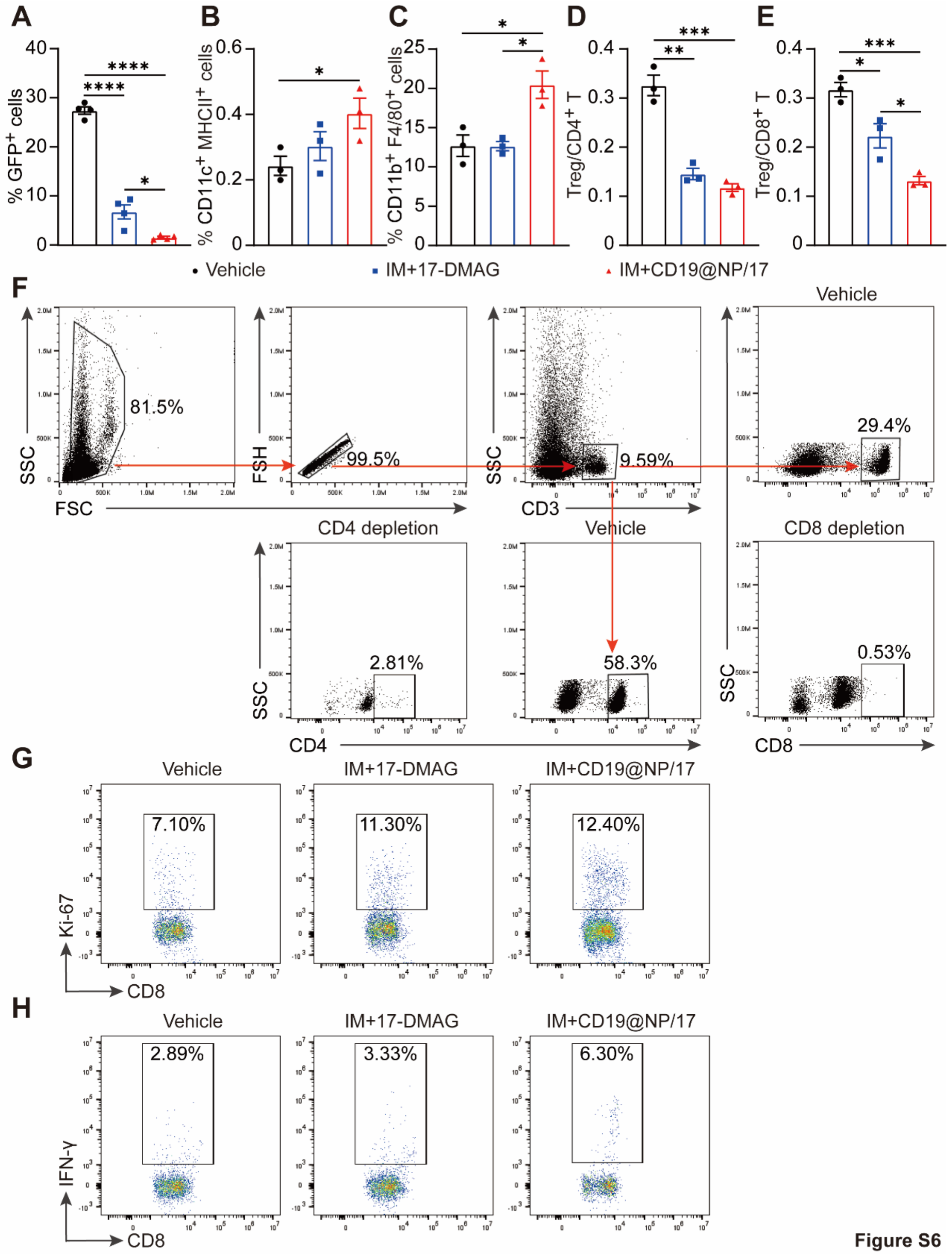

Figure S6. Flow cytometry analysis of immune cell populations following the indicated treatments. (A-E) Splenocytes from BCR-ABL1⁺ B-ALL mice (n = 3) treated as indicated were processed for flow cytometry to assess changes in the percentages of various immune cell populations. Bar graphs show the percentages of GFP⁺ leukemia

cells (A), dendritic cells (DCs) (B), macrophages (C) and the ratios of Treg/CD4$^+$ T cells (D) and Treg/CD8$^+$ T cells (E). (F) Flow cytometry gating steps of antibody depletion experiment. The two images on the far right illustrating the depletion effect of the CD8 antibody, while the bottom two images depict the depletion effect of the CD4 antibody. The vehicle group consisted of wild-type C57 mice without T cell depletion, serving as the control. (G-H) Anti-CD3ε/CD28-stimulated OVA-specific CD8$^+$ T cells were co-cultured with BCR-ABL1$^+$ B-ALL cells pretreated with the indicated treatments in the presence of OVA peptide. The scatter plots illustrate the percentage of Ki-67$^+$ CD8$^+$ T cells (G) and IFN-γ$^+$ CD8$^+$ T cells (H). Data represent three independent experiments. Error bars indicate mean ± SEM. Statistical significance is indicated as follows: *P < 0.05, **P < 0.01, ***P < 0.001 and ****P < 0.0001.

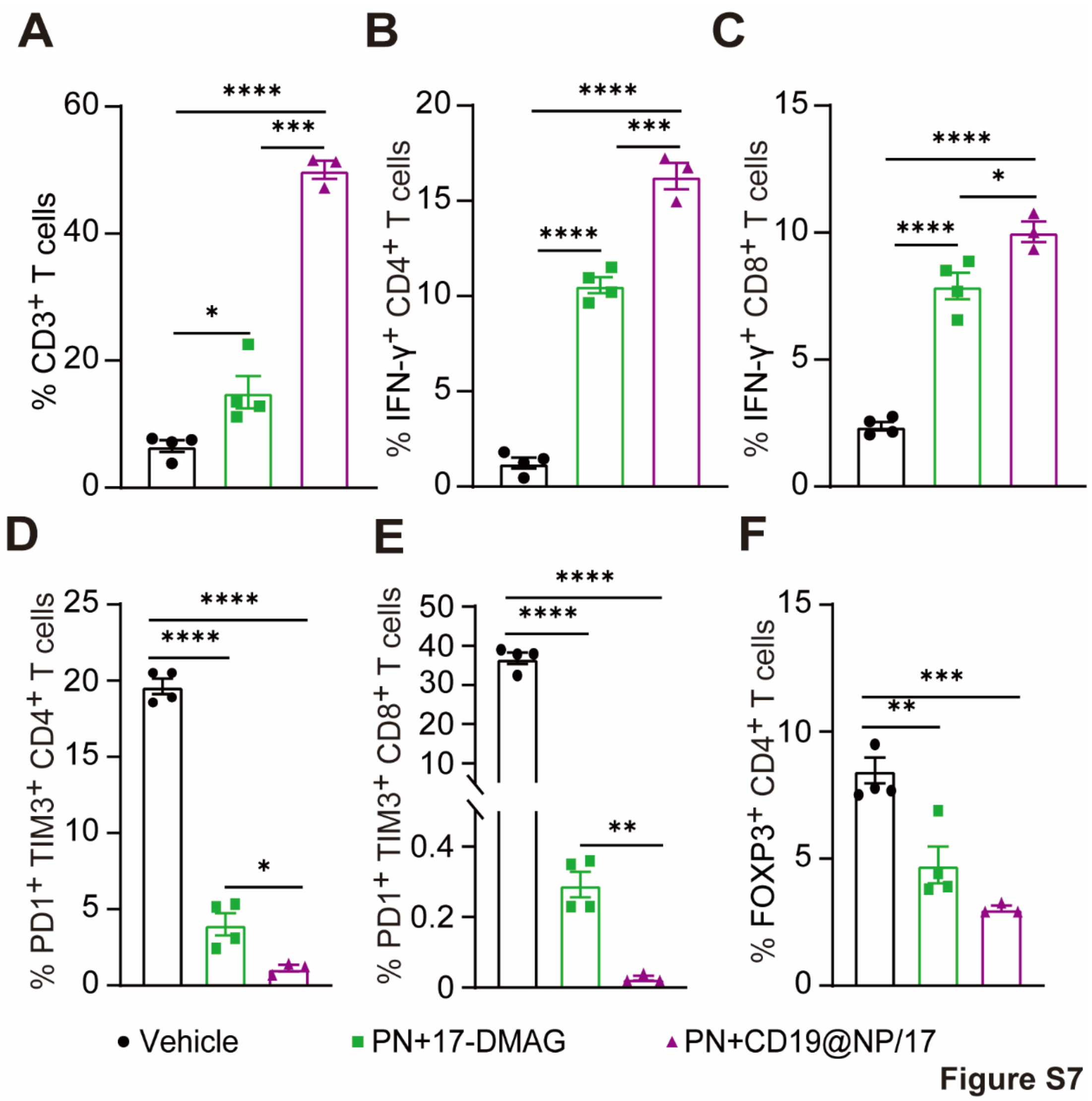

Figure S7. T-cell immune response by CD19@NP/17-DMAG with ponatinib for BCR-ABL1$^+$ B-ALL. (A-F) Analysis of different T cell populations in the spleens of BCR-ABL1$^+$ B-ALL mice following the indicated treatments, when vehicle-treated mice became moribund (n = 4). Bar graphs illustrate the percentages of total CD3$^+$ T cells (A), IFN-γ$^+$ CD4$^+$ T cells (B), IFN-γ$^+$ CD8$^+$ T cells (C), exhausted CD4$^+$ T cells (D), exhausted CD8$^+$ T cells (E) and Treg cells (F). Error bars indicate mean ± SEM. Statistical significance is indicated as follows: *$P < 0.05$, **$P < 0.01$, ***$P < 0.001$ and ****$P < 0.0001$.
